# Maternal Hypertensive Disorder in Pregnancy and Childhood Strabismus in Offspring

**DOI:** 10.1001/jamanetworkopen.2024.23946

**Published:** 2024-07-22

**Authors:** Hui Zhu, Xue You, Yu Jing, Yiyuan Chen, Yangqian Jiang, Yuan Lin, Tao Jiang, Rui Qin, Hong Lv, Qun Lu, Cong Liu, Xin Xu, Yuxin Liu, Tianyu Sun, Mengting Jiang, Bo Xu, Xiumei Han, Jiaping Chen, Yue Jiang, Xiaoyu Liu, Kun Zhou, Guangfu Jin, Hongxia Ma, Zhibin Hu, Hu Liu, Jiangbo Du

**Affiliations:** 1 Department of Ophthalmology, The First Affiliated Hospital with Nanjing Medical University, Nanjing, China; 2 State Key Laboratory of Reproductive Medicine, Nanjing Medical University, Nanjing, China; 3 Department of Epidemiology, Center for Global Health, School of Public Health, Nanjing Medical University, Nanjing, China; 4 Department of Maternal, Child and Adolescent Health, School of Public Health, Nanjing Medical University, Nanjing, China; 5 State Key Laboratory of Reproductive Medicine (Suzhou Centre), The Affiliated Suzhou Hospital of Nanjing Medical University, Suzhou Municipal Hospital, Gusu School, Nanjing Medical University, Suzhou, China; 6 Department of Biostatistics, School of Public Health, Nanjing Medical University, Nanjing, China

## Abstract

**Importance:**

Maternal hypertensive disorder in pregnancy (HDP) might affect ocular health in offspring; however, its association with strabismus remains unclear.

**Objective:**

To examine the association of maternal HDP with overall and type-specific strabismus in offspring.

**Design, Setting, and Participants:**

In the Jiangsu Birth Cohort study, a population-based study in China, pregnant women were recruited from April 24, 2014, to November 30, 2018. A total of 6195 offspring had maternal HDP diagnosis information, of whom 3078 were excluded due to having no information on ocular alignment or due to having ocular diseases other than strabismus or refractive error. Offspring underwent ocular examinations at 3 years of age, completed May 21, 2022. Data were analyzed from May 28, 2022, through December 15, 2023.

**Exposure:**

Maternal HDP, categorized into hypertension and preeclampsia or with blood pressure (BP) well controlled (systolic BP, <130; diastolic BP, <80 mm Hg) and poorly controlled (systolic BP, ≥130; diastolic BP, ≥80 mm Hg).

**Main Outcomes and Measures:**

The primary outcome was the incidence of strabismus in offspring. Poisson generalized linear mixed models were used to estimate the association between maternal HDP and strabismus.

**Results:**

Among the included 3117 children (mean [SD] age, 36.30 [0.74] months; 1629 boys [52.3%]), 143 (4.6%) were exposed to maternal HDP and 368 (11.8%) had strabismus. Offspring exposed to maternal HDP had an 82% increased risk of overall strabismus (relative risk [RR], 1.82 [95% CI, 1.21-2.74]), an 82% increased risk of exophoria (RR, 1.82 [95% CI, 1.11-3.00]), and a 136% increased risk of intermittent exotropia (RR, 2.36 [95% CI, 1.13-4.93]) compared with unexposed offspring. When considering the type of maternal HDP, the risk for all strabismus was high for offspring exposed to preeclampsia (RR, 2.38 [95% CI, 1.39-4.09]) compared with unexposed offspring. When considering the BP control level of maternal HDP, the risk for all strabismus was high for offspring born to mothers with HDP and poorly controlled BP (RR, 2.07 [95% CI, 1.32-3.24]) compared with unexposed offspring.

**Conclusions and Relevance:**

These findings suggest that maternal HDP is associated with an increased risk of offspring strabismus. Early screening of strabismus might be recommended for offspring with maternal HDP. Further exploration of the underlying mechanism of the association between HDP and strabismus is warranted.

## Key Points

**Question:**

Is there an association between maternal hypertensive disorder of pregnancy (HDP) and childhood strabismus in offspring?

**Findings:**

In this cohort study that included 3117 children, offspring born to mothers with HDP had an 82% higher overall risk of strabismus at 3 years of age, and offspring with maternal preeclampsia and poorly controlled blood pressure had the highest risk.

**Meaning:**

These findings suggest that early screening of strabismus might be recommended for offspring born to mothers with HDP, especially those with maternal preeclampsia or poorly controlled blood pressure.

## Teaser

This cohort study investigates the association of maternal hypertensive disorder in pregnancy with overall and type-specific strabismus in offspring at 3 years of age.

## Introduction

Strabismus, which is defined as a deviation from ideal ocular alignment, is one of the most common childhood ocular disorders. It can cause amblyopia, impaired appearance, and psychosocial problems.^1^ If the deviation is absent when both eyes are open for viewing, it is considered latent strabismus; otherwise, it is considered manifest strabismus. The prevalence rate ranges from 4% to 58.3% for latent strabismus^2,3,4,5,6,7^ and from 0.8% to 5.7% for manifest strabismus.^8,9,10,11,12,13^ The etiology and pathogenesis of childhood strabismus remain to be elucidated.^14^ Previous studies have found that perinatal factors, including gestational weeks of age,^15,16,17,18^ birth weight,^15,16,19^ Apgar score,^20^ congenital abnormality,^21^ and parental exposures such as maternal smoking and alcohol consumption during pregnancy,^17,22,23^ are associated with childhood strabismus. These findings imply that aberrant fetal growth and suboptimal intrauterine environments may contribute to the onset of strabismus.

Hypertensive disorder in pregnancy (HDP) is one of the most common cardiometabolic disorders during pregnancy, affecting 5% to 10% of pregnancies worldwide, leading to aberrant fetal growth and intrauterine oxidative stress.^24^ Growing evidence^25,26,27,28,29,30^ has shown that maternal HDP might be related to ocular abnormalities and diseases in offspring, including narrower retinal microvasculature, thinner retina, larger optic cup, refractive error, retinopathy of prematurity, and amblyopia. Some studies^16,21,29,30^ have explored the association of maternal HDP with offspring strabismus, but the findings were preliminary and inconsistent. Recent evidence also suggests that maternal diabetes in pregnancy (DIP) is associated with offspring refractive error.^31^ Therefore, the impact of maternal HDP complicated with maternal DIP on offspring strabismus warrants investigation.

In this prospective birth cohort study, we investigated the association of maternal HDP with overall and type-specific strabismus in offspring at 3 years of age. We further evaluated whether the association differed by the type of HDP and level of blood pressure (BP) control and whether the risk increased when HDP was complicated with DIP.

## Methods

This cohort study was approved by the Human Research Ethics Committee of Nanjing Medical University, and written informed consent was obtained from all participants. The study followed the Strengthening the Reporting of Observational Studies in Epidemiology (STROBE) reporting guideline.

### Study Design and Population

The Jiangsu Birth Cohort study, a subset of the China National Birth Cohort study, aims to assess the influence of perinatal and early life exposure on child health in Jiangsu, China.^32^ In brief, women who were in the first trimester of pregnancy after spontaneous conception or receiving assisted reproductive technology (ART) treatment were recruited, and their children were followed up regularly.

This study included children born in Nanjing who fulfilled the following criteria: (1) singleton or twin live birth, (2) having information on maternal HDP diagnosis, and (3) undergoing ocular examinations at 33 to 39 months of age. From April 24, 2014, to November 30, 2018, 6195 live births had maternal HDP diagnosis information, of whom 3078 were excluded due to having no information on ocular alignment (n = 2978) or having other ocular diseases than strabismus or refractive error (n = 100). Comparisons of characteristics between the included and excluded offspring and their parents are provided in eTable 1 in Supplement 1.

### Maternal HDP Exposure

Exposure data were acquired from electronic medical records (EMR), including HDP, BP during labor, and DIP. Codes from the *International Statistical Classification of Diseases, Tenth Revision*, used to identify different types of maternal HDP are provided in eTable 2 in Supplement 1. Maternal HDP was divided into hypertension (including chronic hypertension and gestational hypertension) and preeclampsia, as well as well-controlled BP (systolic blood pressure [SBP] <130 mm Hg and diastolic blood pressure [DBP] <80 mm Hg) and poorly controlled BP (SBP ≥130 mm Hg or DBP ≥80 mm Hg) during labor.

### Assessment of Strabismus Outcome

To assess ocular alignment, the Hirschberg light reflex test was performed at a distance of 33 cm, followed by cover-uncover test and alternate cover test at distances of both 33 cm and 6 m. Ocular motility was examined at 9 diagnostic positions of gaze. Strabismus was defined as the presence of any latent or manifest ocular deviation and classified according to the primary direction of the deviation. If the manifest deviation was constant at both near and distance fixation, it was considered constant; otherwise, it was intermittent.

### Covariates

Perinatal information on parents and offspring was extracted from questionnaires and the EMR, including maternal age, maternal prepregnancy body mass index (BMI [calculated as weight in kilograms divided by height in meters squared]) (<24 or ≥24), parity (primipara or multipara), maternal smoking or alcohol consumption during pregnancy (yes or no), maternal educational level (≤12 or >12 years), maternal residence (city or country), household annual income in Chinese yuan (<¥100 000 [<$13 792], ¥100 000-¥200 000 [$13 792-$27 583], or >¥200 000 [>$27 583]), paternal age, paternal hypertension or diabetes before pregnancy (yes or no), mode of conception (ART or spontaneous), singleton (yes or no), mode of delivery (cesarean or natural delivery), gestational week at birth, birth weight, offspring Apgar score at 1 and 5 minutes (≤7 or >7), offspring sex (male or female), and offspring congenital abnormality (yes or no). Offspring head circumference was measured using ultrasonography during late pregnancy and classified as less than 10th, 10th to 90th, and greater than 90th percentile according to the International Fetal and Newborn Growth Consortium for the 21st Century standards.^33^ Offspring age was calculated according to the date of ocular examinations and birth date. Offspring refractive error was detected by refractive screening at 3 years of age and defined as the presence of any of the following conditions: (1) spherical equivalent refraction (SER) of less than −1.25 diopters (D) in either eye; (2) SER of greater than 2.5 D in either eye; (3) intereye difference in SER of greater than 1.0 D; and (4) cylindrical refraction of greater than 1.75 D in either eye.^34^ Data on race and ethnicity were not collected. Follow-up was completed on May 21, 2022.

### Statistical Analysis

Data were analyzed from May 28, 2022, through December 15, 2023. Poisson generalized linear mixed model with a random intercept for each mother was used to estimate the relative risks (RRs) and 95% CIs for the association between maternal HDP and offspring strabismus, given the nonindependence of observations from twins.^35^ Further, we examined whether the association differed by the type of HDP and level of BP control and whether the risk increased when HDP was complicated with DIP.

A directed acyclic graph (eFigure in Supplement 1) was developed to inform our staged modeling approach.^36^ Model 1 was unadjusted. Model 2 was adjusted for 9 confounders, including maternal age, paternal age, maternal educational level, maternal residence, household annual income, mode of conception, singleton, offspring sex, and offspring age. Model 3 was additionally adjusted for 4 covariates suspected to lie on the causal pathway, including mode of delivery, gestational week of age, birth weight, and offspring refractive error.

Several sensitivity analyses were performed by limiting consideration to a specific subgroup of participants. Since maternal alcohol consumption or smoking during pregnancy, abnormal offspring Apgar score, offspring congenital abnormality, and offspring head circumference were not present or measured in many children, these covariates were evaluated only in sensitivity analyses. These analyses were restricted to children without exposure to maternal alcohol consumption during pregnancy, without exposure to maternal smoking during pregnancy, with normal Apgar scores (defined as >7 at both 1 and 5 minutes), without congenital abnormality, and with normal head circumference (10th-90th percentile). Another 5 sensitivity analyses were performed that were restricted to children born at full term with normal birth weight (defined as ≥2.5 kg), conducted after excluding children with exposure to paternal hypertension or diabetes before pregnancy, restricted to children born to mothers with prepregnancy BMI of less than 24, restricted to children born to primipara, and conducted after propensity score matching between the HDP and non-HDP groups according to all covariates in model 3, using 1:4 nearest neighbor matching without replacement.

Information on missing covariate data is given in eTable 3 in Supplement 1. We handled missing data (<10% of participants) using complete case analysis and compared characteristics between offspring with and without complete data (eTables 4 and 5 in Supplement 1). All analyses were conducted using R software, version 4.1.2 (R Project for Statistical Computing), with a 2-sided *P* < .05 considered statistically significant.

## Results

Among the 3117 children (mean [SD] age, 36.30 [0.74] months; 1488 girls [47.7%] and 1629 boys [52.3%]) born to 3005 mothers (mean [SD] age, 30.40 [3.76] years; mean [SD] prepregnancy BMI, 21.38 [3.01]) included in the analysis, 143 (4.6%) were exposed to maternal HDP, including 78 with maternal hypertension and 65 with maternal preeclampsia. Among these 143 children, maternal BP was well controlled in 33, poorly controlled in 103, and unknown in 7. Compared with the non-HDP group, the HDP group had older parents; a higher maternal prepregnancy BMI; more frequent conception through ART, twins, cesarean delivery, and maternal urban residence; and lower gestational age, birth weight, household income, and maternal educational level (Table 1).

**Table 1.** Characteristics of Parents and of Offspring With and Without Maternal HDP

Characteristic	Study group^a^	
	With maternal HDP (n = 143)	Without maternal HDP (n = 2974)
**Maternal characteristics**		
Age, mean (SD), y	31.36 (3.65)	30.35 (3.76)
Prepregnancy BMI		
<24	84 (59.2)	2500 (84.4)
≥24	58 (40.8)	461 (15.6)
Alcohol consumption		
No	139 (99.3)	2904 (98.3)
Yes	1 (0.7)	50 (1.7)
Smoking		
No	139 (99.3)	2939 (99.5)
Yes	1 (0.7)	15 (0.5)
DIP		
No	103 (72.0)	2202 (74.0)
Yes	40 (28.0)	772 (26.0)
Parity		
Primipara	118 (84.3)	2433 (82.3)
Multipara	22 (15.7)	523 (17.7)
Educational level, y		
≤12	31 (21.8)	281 (9.5)
>12	111 (78.2)	2684 (90.5)
Residence		
Country	11 (7.7)	481 (16.2)
City	131 (92.3)	2493 (83.8)
Household annual income, ¥^b^		
<100 000	62 (43.7)	946 (33.0)
100 000-200 000	67 (47.2)	1279 (44.7)
>200 000	13 (9.2)	638 (22.3)
**Paternal characteristics**		
Age, mean (SD), y	32.78 (5.45)	31.86 (4.66)
Hypertension before pregnancy		
No	143 (100)	2964 (99.7)
Yes	0	10 (0.3)
Diabetes before pregnancy		
No	143 (100)	2968 (99.8)
Yes	0	6 (0.2)
**Offspring characteristics**		
Sex		
Male	79 (55.2)	1550 (52.1)
Female	64 (44.8)	1424 (47.9)
Age, mean (SD), mo	36.27 (0.72)	36.30 (0.74)
Mode of conception		
Spontaneous	85 (59.4)	2535 (85.2)
ART	58 (40.6)	439 (14.8)
Singleton		
Yes	96 (67.1)	2771 (93.2)
No	47 (32.9)	203 (6.8)
Mode of delivery		
Natural delivery	35 (24.5)	1640 (55.3)
Cesarean delivery	108 (75.5)	1325 (44.7)
Gestational age at birth, mean (SD), wk	37.77 (2.21)	38.89 (1.59)
Birth weight, mean (SD), kg	3.07 (0.70)	3.33 (0.47)
Apgar score at 1 min		
>7	137 (100)	2918 (99.6)
≤7	0	11 (0.4)
Apgar score at 5 min		
>7	137 (100)	2894 (100)
≤7	0	0
Congenital abnormality		
No	133 (94.3)	2834 (95.6)
Yes	8 (5.7)	130 (4.4)
Head circumference		
<10th Percentile	3 (4.7)	55 (3.4)
10th-90th Percentile	55 (85.9)	1442 (89.0)
>90th Percentile	6 (9.4)	123 (7.6)
Refractive error		
No	121 (85.8)	2460 (85.5)
Yes	20 (14.2)	417 (14.5)
Strabismus		
No	115 (80.4)	2634 (88.6)
Yes	28 (19.6)	340 (11.4)

Abbreviations: ART, assisted reproductive technology; BMI, body mass index (calculated as weight in kilograms divided by height in meters squared); DIP, diabetes in pregnancy; HDP, hypertensive disorder of pregnancy.

^a^ Unless otherwise indicated, data are expressed as No. (%) of patients. Owing to missing data, denominators may be less than totals in column headings.

^b^ To convert to US$, multiply by 0.14.

Among the 3117 children, 368 (11.8%) were found to have strabismus, including 265 (8.5%) with latent strabismus and 103 (3.3%) with manifest strabismus. Among the 265 children with latent strabismus, 260 (98.1%) had exophoria and 5 (1.9%) had esophoria. Among the 103 children with manifest strabismus, 95 (92.2%) had intermittent exotropia, 4 (3.9%) had constant exotropia, 1 (1.0%) had intermittent esotropia, and 3 (2.9%) had constant esotropia.

Offspring exposed to maternal HDP had an 82% increased risk of overall strabismus compared with unexposed offspring (model 3 RR, 1.82 [95% CI, 1.21-2.74]); this pattern was similar for type-specific strabismus, with an 82% increased risk for exophoria (model 3 RR, 1.82 [95% CI, 1.11-3.00]) and a 136% increased risk for intermittent exotropia (model 3 RR, 2.36 [95% CI, 1.13-4.93]) (Table 2). When considering the type of maternal HDP, the risk for all strabismus was high for offspring exposed to preeclampsia (model 3 RR, 2.38 [95% CI, 1.39-4.09]) compared with unexposed offspring; this trend was apparent in exophoria (model 3 RR for preeclampsia, 2.68 [95% CI, 1.42-5.03]) but not in intermittent exotropia (model 3 RR for preeclampsia, 2.51 [95% CI, 0.87-7.25]) (Table 2).

**Table 2.** Relative Risks for the Association Between Maternal HDP and Overall and Specific Types of Strabismus in Offspring

Outcome by exposure	No. with outcome/total No. (%)	Model 1^a^		Model 2^b^		Model 3^c^	
		RR (95% CI)	*P* value	RR (95% CI)	*P* value	RR (95% CI)	*P* value
**Overall strabismus**							
No maternal HDP	340/2974 (11.4)	1 [Reference]	NA	1 [Reference]	NA	1 [Reference]	NA
Maternal HDP							
Overall	28/143 (19.6)	1.71 (1.17-2.52)	.006	1.71 (1.14-2.57)	.01	1.82 (1.21-2.74)	.004
Hypertension	12/78 (15.4)	1.35 (0.76-2.39)	.31	1.36 (0.76-2.43)	.31	1.41 (0.78-2.54)	.25
Preeclampsia	16/65 (24.6)	2.15 (1.30-3.55)	.003	2.18 (1.27-3.73)	.005	2.38 (1.39-4.09)	.002
**Exophoria**							
No maternal HDP	241/2875 (8.4)	1 [Reference]	NA	1 [Reference]	NA	1 [Reference]	NA
Maternal HDP							
Overall	19/134 (14.2)	1.69 (1.06-2.70)	.03	1.74 (1.06-2.87)	.03	1.82 (1.11-3.00)	.02
Hypertension	7/73 (9.6)	1.14 (0.54-2.43)	.73	1.18 (0.55-2.53)	.66	1.23 (0.57-2.63)	.60
Preeclampsia	12/61 (19.7)	2.35 (1.31-4.19)	.004	2.54 (1.35-4.77)	.004	2.68 (1.42-5.03)	.002
**Intermittent exotropia**							
No maternal HDP	86/2720 (3.2)	1 [Reference]	NA	1 [Reference]	NA	1 [Reference]	NA
Maternal HDP							
Overall	9/124 (7.3)	2.30 (1.16-4.56)	.02	2.05 (0.99-4.24)	.05	2.36 (1.13-4.93)	.02
Hypertension	5/71 (7.0)	2.23 (0.90-5.48)	.08	2.08 (0.83-5.23)	.12	2.26 (0.89-5.76)	.09
Preeclampsia	4/53 (7.5)	2.39 (0.88-6.50)	.09	2.01 (0.70-5.76)	.19	2.51 (0.87-7.25)	.09

Abbreviations: HDP, hypertensive disorder of pregnancy; NA, not applicable; RR, relative risk.

^a^ Unadjusted.

^b^ Adjusted for maternal age, paternal age, maternal educational level, maternal residence, household annual income, mode of conception, singleton, offspring sex, and offspring age.

^c^ Adjusted for maternal age, paternal age, maternal educational level, maternal residence, household annual income, mode of conception, singleton, offspring sex, offspring age, mode of delivery, gestational age at birth, birth weight, and offspring refractive error.

When considering the BP control level of maternal HDP, the risk for all strabismus was high for offspring born to mothers with HDP and poorly controlled BP (model 3 RR, 2.07 [95% CI, 1.32-3.24]) compared with unexposed offspring; this trend was apparent both in exophoria (model 3 RR for poorly controlled BP, 2.13 [95% CI, 1.24-3.65]) and intermittent exotropia (model 3 RR for poorly controlled BP, 2.61 [95% CI, 1.15-5.93]) (Figure). When considering both the type of maternal HDP and level of BP control, the highest risk was observed in offspring exposed to preeclampsia and poorly controlled BP (model 3 RR, 2.45 [95% CI, 1.37-4.39]) (Table 3).

**Figure.**
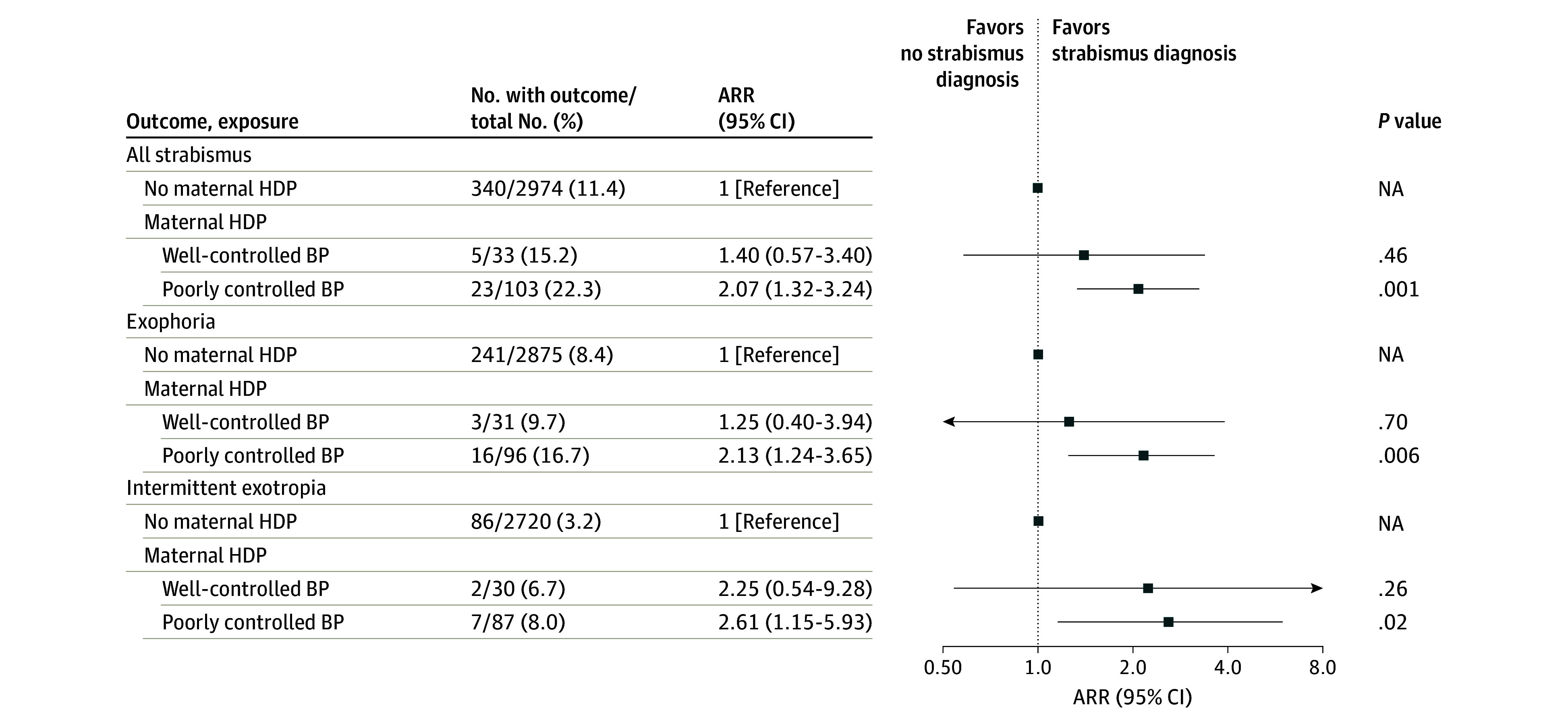
Relative Risks of Offspring Strabismus for Different Blood Pressure (BP) Control Levels of Maternal Hypertensive Disorder of Pregnancy (HDP) Data are from analyses adjusted for covariates in model 3, including maternal age, paternal age, maternal educational level, maternal residence, household annual income, mode of conception, singleton, offspring sex, offspring age, mode of delivery, gestational week at birth, birth weight, and offspring refractive error. ARR indicates adjusted relative risk; NA, not applicable.

**Table 3.** Relative Risks of Overall Strabismus in Offspring When Considering Maternal HDP Based on Type and BP Control

Exposure	No. with outcome/total No. (%)	Model 1^a^		Model 2^b^		Model 3^c^	
		RR (95% CI)	*P* value	RR (95% CI)	*P* value	RR (95% CI)	*P* value
No maternal HDP	340/2974 (11.4)	1 [Reference]	NA	1 [Reference]	NA	1 [Reference]	NA
Hypertension and well-controlled BP	3/24 (12.5)	1.09 (0.35-3.41)	.88	1.16 (0.37-3.65)	.80	1.19 (0.38-3.74)	.77
Hypertension and poorly controlled BP	9/47 (19.1)	1.67 (0.86-3.25)	.13	1.62 (0.83-3.16)	.16	1.71 (0.87-3.34)	.12
Preeclampsia and well-controlled BP	2/9 (22.2)	1.94 (0.48-7.80)	.35	1.84 (0.45-7.42)	.39	1.91 (0.47-7.72)	.37
Preeclampsia and poorly controlled BP	14/56 (25.0)	2.19 (1.28-3.73)	.004	2.21 (1.24-3.96)	.007	2.45 (1.37-4.39)	.003

Abbreviations: BP, blood pressure; HDP, hypertensive disorder of pregnancy; NA, not applicable; RR, relative risk.

^a^ Unadjusted.

^b^ Adjusted for maternal age, paternal age, maternal educational level, maternal residence, household annual income, mode of conception, singleton, offspring sex, and offspring age.

^c^ Adjusted for maternal age, paternal age, maternal educational level, maternal residence, household annual income, mode of conception, singleton, offspring sex, offspring age, mode of delivery, gestational age at birth, birth weight, and offspring refractive error.

No association between maternal DIP and strabismus was found (eTable 6 in Supplement 1). Compared with offspring with neither exposure, the fully adjusted RRs for all strabismus were 1.77 (95% CI, 1.08-2.89) in offspring exposed only to maternal HDP, 0.94 (95% CI, 0.72-1.22) in offspring exposed to only maternal DIP, and 1.83 (95% CI, 0.89-3.73) in offspring exposed to both (Table 4).

**Table 4.** Relative Risks of Overall Strabismus in Offspring When Maternal HDP Is Complicated With Maternal DIP

Exposure	No. with outcome/total No. (%)	Model 1^a^		Model 2^b^		Model 3^c^	
		RR (95% CI)	*P* value	RR (95% CI)	*P* value	RR (95% CI)	*P* value
Neither^d^	252/2202 (11.4)	1 [Reference]	NA	1 [Reference]	NA	1 [Reference]	NA
Only HDP	20/103 (19.4)	1.70 (1.08-2.68)	.02	1.67 (1.02-2.72)	.04	1.77 (1.08-2.89)	.02
Only DIP	88/772 (11.4)	1.00 (0.78-1.27)	.98	0.97 (0.75-1.25)	.79	0.94 (0.72-1.22)	.65
Both	8/40 (20.0)	1.75 (0.86-3.53)	.12	1.74 (0.85-3.54)	.13	1.83 (0.89-3.73)	.10

Abbreviations: DIP, diabetes in pregnancy; HDP, hypertensive disorder of pregnancy; NA, not applicable; RR, relative risk.

^a^ Unadjusted.

^b^ Adjusted for maternal age, paternal age, maternal educational level, maternal residence, household annual income, mode of conception, singleton, offspring sex, and offspring age.

^c^ Adjusted for maternal age, paternal age, maternal educational level, maternal residence, household annual income, mode of conception, singleton, offspring sex, offspring age, mode of delivery, gestational age at birth, birth weight, and offspring refractive error.

^d^ Indicates no HDP or DIP.

When we restricted the analysis to children without exposure to maternal alcohol consumption or smoking during pregnancy, with normal Apgar scores at 1 and 5 minutes, without congenital abnormality, with normal head circumference, or born to primipara, we obtained similar results to those of the main analyses (eTables 7-12 in Supplement 1). We observed almost identical results to those of the main analyses when we restricted the analysis to children born full-term with normal birth weight (eTable 13 in Supplement 1), after excluding children with exposure to paternal hypertension or diabetes before pregnancy (eTable 14 in Supplement 1), or to children born to mothers with prepregnancy BMI of less than 24 (eTable 15 in Supplement 1). In addition, results of analyses after propensity score matching were consistent with those of the main analyses (eTable 16 in Supplement 1).

## Discussion

In this cohort study, offspring born to mothers with HDP were found to have increased risks of overall and type-specific strabismus, including exophoria and intermittent exotropia, at 3 years of age. The highest risk was observed in offspring born to mothers with preeclampsia and poorly controlled BP.

The origins of strabismus are widely considered to be abnormalities in binocular vision.^14,37^ Binocular vision is a complex process in the brain to combine 2 images—1 from each eye—into a single image that is formed under the conditions of good vision, normal ocular motility, and a well-developed brain.^14,37^ Maternal HDP might increase the risk of strabismus by damaging binocular vision through the following mechanisms. First, placental hypoxia related to HDP may lead to permanent structural changes in the fetal brain.^38^ Animal experiments have shown that even brief periods of hypoxia can result in neuronal death, white matter damage, and reduced growth of neural processes in the fetus.^39^ Second, HDP, especially preeclampsia, can lead to increased inflammation and exaggerated oxidative stress in fetal circulation, which may damage fetal neurons,^40,41^ leading to negative effects on the development of the brain in offspring.^42,43,44,45^ Third, the oxidative stress led by HDP might cause degeneration of photoreceptors and other cells in retina.^46^ In one cohort study, children exposed to maternal HDP were found to have thinner retina,^28^ supporting the adverse influence of HDP on retina development. Fourth, maternal HDP might also impact offspring vision through inducing refractive error.^25^ Finally, maternal HDP would increase the risk of cesarean delivery,^47^ preterm birth,^48^ and low birth weight^49^ in offspring, all of which have potential adverse effects on the development of the brain and vision.^50,51,52^

Four previous studies^16,21,29,30^ have explored the association between maternal HDP and offspring strabismus but with different methods. A cross-sectional study of 14 980 children aged 3 years^16^ found no association between maternal increased BP during pregnancy and parental reported strabismus of offspring; however, increased BP is not the same as HDP, and some types of strabismus, especially latent strabismus and intermittent exotropia, are not easily recognized by parents due to small change in appearance. One cohort study (n = 96 842)^21^ showed that offspring exposed to maternal preeclampsia had a slightly higher risk of manifest strabismus. The influence of other HDP types was not analyzed, and the outcome was obtained by reviewing ophthalmological records in this study, which might underestimate the strabismus rate. One small study of 78 preterm children aged 5 years^29^ failed to find any difference in the rates of latent and manifest strabismus, obtained through ocular examinations, between children born to mothers with and without preeclampsia. While another cohort study (n = 1125) also using ocular examinations to detect strabismus^30^ found that maternal chronic hypertension was related to an increased risk of exotropia at 20 years old, some offspring with strabismus might have been treated during childhood and therefore were regarded as normal, because strabismus usually develops during early childhood. To our knowledge, our study is the first prospective cohort study to evaluate the association of maternal HDP with overall and type-specific strabismus in early childhood, with the outcomes measured by ocular examinations and HDP classified into different types and levels of BP control. As mentioned above, maternal HDP might increase the risk of strabismus in offspring through its influence on refractive error, cesarean delivery, gestational week of age, and birth weight. We applied a staged modeling approach to clarify the role of these possible intermediate factors, and our findings indicate the association between maternal HDP and strabismus may be independent of these factors.

The findings of the current study suggest that preeclampsia was associated with a higher risk of offspring strabismus than hypertension. These findings are understandable given that preeclampsia is a multisystemic disorder that targets several vital organs and is characterized by proteinuria or evidence of systemic disease, rather than just high BP.^53^ We also found that the increased risk of strabismus is more pronounced among offspring exposed to maternal HDP with poorly controlled BP than well-controlled BP, indicating the importance of controlling BP. Mothers with SBP of 130 to 139 mm Hg or DBP of 80 to 89 mm Hg have been found to have increased risk of preterm birth in offspring.^54,55^ Similarly, a higher risk of cardiovascular mortality was observed in pregnant women with DBP of at least 80 mm Hg than those with DBP of less than 80 mm Hg in a cohort study.^56^

Maternal HDP and DIP might have shared pathological processes.^24^ One prospective study found that maternal DIP was associated with a high risk of refractive error in offspring,^31^ indicating that DIP might also influence offspring ocular health. However, we failed to find associations between maternal DIP and offspring strabismus or an increased risk of offspring strabismus when maternal HDP was complicated with DIP. More research is needed to clarify the reason for this discrepancy.

### Strengths and Limitations

The primary strength of our study is the prospective cohort design, allowing for collecting accurate data on exposure, outcome, and covariates. Ocular examinations were performed by ophthalmologists who were unaware of maternal HDP diagnosis. Possible influence of various covariates has been adjusted in multivariable models or evaluated in sensitivity analyses.

Several limitations should also be noted. First, residual confounding might exist, such as postnatal environmental exposures and family history of strabismus. Second, other strabismus types could not be analyzed, since no children exposed to maternal HDP had strabismus other than exophoria and intermittent exotropia. Larger sample studies are warranted to explore the influence of maternal HDP on other strabismus types. Third, although chronic hypertension and gestational hypertension might have different associations with offspring strabismus, we combined chronic hypertension and gestational hypertension for our analyses due to the small sample size with chronic hypertension. Fourth, we categorized maternal HDP into different levels of BP control according to BP measured during labor; however, BP might vary from pregnancy to delivery, and more frequent measurements of BP at different time points might be better. Fifth, the number of children with exposure to maternal HDP and strabismus was relatively small, limiting direct comparisons between different HDP types, levels of BP control, or their combinations. However, the trend shown in exploratory analyses supported our findings (eTable 17 in Supplement 1). Sixth, there might be bias due to the exclusion of offspring with missing outcomes data. Seventh, ascertainment bias might be caused by the differences of birth weight and gestational age between offspring with and without complete data, although we included them in model 3 and performed a sensitivity analysis among children born full-term with normal birth weight. Eighth, coding errors might exist in the EMR, which cause possible misclassification of exposure. However, this misclassification is likely to be nondifferential and weaken the risk estimates.

## Conclusions

The findings of this cohort study suggest that maternal HDP was associated with increased risks of overall and type-specific strabismus, including exophoria and intermittent exotropia, in offspring at 3 years of age. Offspring born to mothers with HDP might be recommended for early screening of strabismus, especially those with maternal preeclampsia or poorly controlled BP. The underlying mechanism of the association between maternal HDP and strabismus in offspring warrants further exploration.

## Notes

Supplement 1. **eTable 1.** Comparisons of Characteristics Between the Included and Excluded Offspring and Their Parents **eTable 2.** The *ICD-10* Codes Used to Identify Different Types of Maternal HDP **eTable 3.** Missing Data for Covariates **eTable 4.** Comparisons of Characteristics Between Offspring With Complete and Incomplete Data of Covariates Adjusted in Model 2 **eTable 5.** Comparisons of Characteristics Between Offspring With Complete and Incomplete Data of Covariates Adjusted in Model 3 **eTable 6.** Relative Risks for the Association Between Maternal DIP and Overall and Specific Types of Strabismus in Offspring **eTable 7.** Relative Risks for the Association Between Maternal HDP and Overall and Specific Types of Strabismus Restricted to Offspring Without Exposure to Maternal Alcohol Consumption During Pregnancy **eTable 8.** Relative Risks for the Association Between Maternal HDP and Overall and Specific Types of Strabismus Restricted to Offspring Without Exposure to Maternal Smoking During Pregnancy **eTable 9.** Relative Risks for the Association Between Maternal HDP and Overall and Specific Types of Strabismus Restricted to Offspring With Normal Apgar Score **eTable 10.** Relative Risks for the Association Between Maternal HDP and Overall and Specific Types of Strabismus Restricted to Offspring Without Congenital Abnormality **eTable 11.** Relative Risks for the Association Between Maternal HDP and Overall and Specific Types of Strabismus Restricted to Offspring With Normal Head Circumference **eTable 12.** Relative Risks for the Association Between Maternal HDP and Overall and Specific Types of Strabismus Restricted to Offspring Born to Primipara **eTable 13.** Relative Risks for the Association Between Maternal HDP and Overall and Specific Types of Strabismus Restricted to Term-Born Children of Normal Birth Weight **eTable 14.** Relative Risks for the Association Between Maternal HDP and Overall and Specific Types of Strabismus After Excluding Children With Exposure to Paternal Hypertension or Diabetes Before Pregnancy **eTable 15.** Relative Risks for the Association Between Maternal HDP and Overall and Specific Types of Strabismus Restricted to Children Born to Mothers With Prepregnancy BMI <24 **eTable 16.** Relative Risks for the Association Between Maternal HDP and Overall and Specific Types of Strabismus After PSM **eTable 17.** Direct Comparisons of Overall Strabismus Risk in Offspring Between Different HDP Types, BP Control Levels, or Their Combinations **eFigure.** Directed Acyclic Graph Documenting Assumptions About the Association Between Covariates, Exposure, and Outcome

Supplement 2.
Nonauthor Collaborators

Supplement 3.
Data Sharing Statement
