# Supplementary material for: Maternal Hypertensive Disorder in Pregnancy and Childhood Strabismus in Offspring
Source: JAMA Netw Open. 2024 Jul 22;7(7):e2423946. doi: 10.1001/jamanetworkopen.2024.23946 (PMC11265127; doi:10.1001/jamanetworkopen.2024.23946)
Supplement: Supplement 1. — eTable 1. Comparisons of Characteristics Between the Included and Excluded Offspring and Their Parents eTable 2. The ICD-10 Codes Used to Identify Different Types of Maternal HDP eTable 3. Missing Data for Covariates eTable 4. Comparisons of Characteristics Between Offspring With Complete and Incomplete Data of Covariates Adjusted in Model 2 eTable 5. Comparisons of Characteristics Between Offspring With Complete and Incomplete Data of Covariates Adjusted in Model 3 eTable 6. Relative Risks for the Association Between Maternal DIP and Overall and Specific Types of Strabismus in Offspring eTable 7. Relative Risks for the Association Between Maternal HDP and Overall and Specific Types of Strabismus Restricted to Offspring Without Exposure to Maternal Alcohol Consumption During Pregnancy eTable 8. Relative Risks for the Association Between Maternal HDP and Overall and Specific Types of Strabismus Restricted to Offspring Without Exposure to Maternal Smoking During Pregnancy eTable 9. Relative Risks for the Association Between Maternal HDP and Overall and Specific Types of Strabismus Restricted to Offspring With Normal Apgar Score eTable 10. Relative Risks for the Association Between Maternal HDP and Overall and Specific Types of Strabismus Restricted to Offspring Without Congenital Abnormality eTable 11. Relative Risks for the Association Between Maternal HDP and Overall and Specific Types of Strabismus Restricted to Offspring With Normal Head Circumference eTable 12. Relative Risks for the Association Between Maternal HDP and Overall and Specific Types of Strabismus Restricted to Offspring Born to Primipara eTable 13. Relative Risks for the Association Between Maternal HDP and Overall and Specific Types of Strabismus Restricted to Term-Born Children of Normal Birth Weight eTable 14. Relative Risks for the Association Between Maternal HDP and Overall and Specific Types of Strabismus After Excluding Children With Exposure to Paternal Hypertension or Diabetes Before Pregn [file jamanetwopen-e2423946-s001.pdf]

# Supplementary Online Content

Zhu H, You X, Jing Y, et al; China National Birth Cohort Study Group. Maternal hypertensive disorder in pregnancy and childhood strabismus in offspring. *JAMA Netw Open*. 2024;7(7):e2423946. doi:10.1001/jamanetworkopen.2024.23946

**eTable 1.** Comparisons of Characteristics Between the Included and Excluded Offspring and Their Parents
**eTable 2.** The ICD-10 Codes Used to Identify Different Types of Maternal HDP
**eTable 3.** Missing Data for Covariates
**eTable 4.** Comparisons of Characteristics Between Offspring With Complete and Incomplete Data of Covariates Adjusted in Model 2
**eTable 5.** Comparisons of Characteristics Between Offspring With Complete and Incomplete Data of Covariates Adjusted in Model 3
**eTable 6.** Relative Risks for the Association Between Maternal DIP and Overall and Specific Types of Strabismus in Offspring
**eTable 7.** Relative Risks for the Association Between Maternal HDP and Overall and Specific Types of Strabismus Restricted to Offspring Without Exposure to Maternal Alcohol Consumption During Pregnancy
**eTable 8.** Relative Risks for the Association Between Maternal HDP and Overall and Specific Types of Strabismus Restricted to Offspring Without Exposure to Maternal Smoking during Pregnancy
**eTable 9.** Relative Risks for the Association Between Maternal HDP and Overall and Specific Types of Strabismus Restricted to Offspring With Normal Apgar Score
**eTable 10.** Relative Risks for the Association Between Maternal HDP and Overall and Specific Types of Strabismus Restricted to Offspring Without Congenital Abnormality
**eTable 11.** Relative Risks for the Association Between Maternal HDP and Overall and Specific Types of Strabismus Restricted to Offspring With Normal Head Circumference
**eTable 12.** Relative Risks for the Association Between Maternal HDP and Overall and Specific Types of Strabismus Restricted to Offspring Born to Primipara
**eTable 13.** Relative Risks for the Association Between Maternal HDP and Overall and Specific Types of Strabismus Restricted to Term-born Children of Normal Birth Weight
**eTable 14.** Relative Risks for the Association Between Maternal HDP and Overall and Specific Types of Strabismus after Excluding Children With Exposure to Paternal Hypertension or Diabetes before Pregnancy
**eTable 15.** Relative Risks for the Association Between Maternal HDP and Overall and Specific Types of Strabismus Restricted to Children Born to Mothers With Prepregnancy BMI <24
**eTable 16.** Relative Risks for the Association Between Maternal HDP and Overall and Specific Types of Strabismus after PSM
**eTable 17.** Direct Comparisons of Overall Strabismus Risk in Offspring Between Different HDP Types, BP Control Levels, or Their Combinations

© 2024 Zhu H et al. *JAMA Network Open*.

**eFigure.** Directed Acyclic Graph Documenting Assumptions About the Association Between Covariates, Exposure, and Outcome

This supplementary material has been provided by the authors to give readers additional information about their work.

© 2024 Zhu H et al. *JAMA Network Open*.

**eTable 1. Comparisons of Characteristics Between the Included and Excluded Offspring and Their Parents**

| Characteristic | Included (n=3117) | Excluded (n=3067) | *P* value[a] |
|---|---|---|---|
| HDP, No. (%) | | | |
| No | 2974 (95.4) | 2864 (93.4) | 0.001 |
| Yes | 143 (4.6) | 203 (6.6) | |
| Maternal age, mean (SD), year | 30.40 (3.76) | 30.67 (3.91) | 0.005 |
| Maternal prepregnancy BMI, No. (%) | | | |
| <24 kg/m$^2$ | 2584 (83.3) | 2511 (82.1) | 0.23 |
| ≥24 kg/m$^2$ | 519 (16.7) | 548 (17.9) | |
| Maternal alcohol consumption, No. (%) | | | |
| No | 3043 (98.4) | 2993 (98.1) | 0.46 |
| Yes | 51 (1.6) | 59 (1.9) | |
| Maternal smoking, No. (%) | | | |
| No | 3078 (99.5) | 3026 (99.1) | 0.12 |
| Yes | 16 (0.5) | 27 (0.9) | |
| Maternal DIP, No. (%) | | | |
| No | 2305 (73.9) | 2278 (74.3) | 0.79 |
| Yes | 812 (26.1) | 789 (25.7) | |
| Parity, No. (%) | | | |
| Primipara | 2551 (82.4) | 2497 (81.5) | 0.38 |
| Multipara | 545 (17.6) | 567 (18.5) | |
| Maternal education level, No. (%) | | | |
| ≤12 years | 312 (10.0) | 485 (15.8) | <.001 |
| >12 years | 2795 (90.0) | 2580 (84.2) | |
| Maternal residence, No. (%) | | | |
| Country | 492 (15.8) | 504 (16.4) | 0.51 |
| City | 2624 (84.2) | 2563 (83.6) | |
| Household annual income, No. (%) | | | |
| <100000 CNY | 1008 (33.5) | 1051 (35.3) | |
| 100000-200000 CNY | 1346 (44.8) | 1200 (40.3) | 0.001 |
| >200000 CNY | 651 (21.7) | 725 (24.4) | |
| Paternal age, mean (SD), year | 31.90 (4.70) | 32.21 (4.81) | 0.01 |
| Paternal hypertension before pregnancy, No. (%) | | | |
| No | 3107 (99.7) | 3057 (99.7) | >.99 |
| Yes | 10 (0.3) | 10 (0.3) | |
| Paternal diabetes before pregnancy, No. (%) | | | |
| No | 3111 (99.8) | 3060 (99.8) | 0.98 |
| Yes | 6 (0.2) | 7 (0.2) | |
| Mode of conception, No. (%) | | | |
| Spontaneously conceived | 2620 (84.1) | 2292 (74.7) | <.001 |
| ART | 497 (15.9) | 775 (25.3) | |
| Singleton, No. (%) | | | |

| | | | |
|---|---|---|---|
| Yes | 2867 (92.0) | 2682 (87.4) | <.001 |
| No | 250 (8.0) | 385 (12.6) | |
| Mode of delivery, No. (%) | | | |
| Natural delivery | 1675 (53.9) | 1405 (46.3) | <.001 |
| Cesarean section | 1433 (46.1) | 1632 (53.7) | |
| Gestational week at birth, mean (SD), week | 38.84 (1.64) | 38.50 (1.96) | <.001 |
| Birth weight, mean (SD), kilogram | 3.31 (0.48) | 3.26 (0.54) | <.001 |
| Apgar score at 1 minute, No. (%) | | | |
| >7 | 3055 (99.6) | 2885 (99.6) | 0.73 |
| ≤7 | 11 (0.4) | 13 (0.4) | |
| Apgar score at 5 minutes, No. (%) | | | |
| >7 | 3031 (100.0) | 2777 (99.8) | 0.06 |
| ≤7 | 0 (0.0) | 5 (0.2) | |
| Offspring sex, No. (%) | | | |
| Male | 1629 (52.3) | 1628 (53.1) | 0.54 |
| Female | 1488 (47.7) | 1439 (46.9) | |
| Offspring congenital abnormality, No. (%) | | | |
| No | 2967 (95.6) | 2888 (94.6) | 0.08 |
| Yes | 138 (4.4) | 166 (5.4) | |
| Offspring head circumference, No. (%) | | | |
| <10$^{th}$ centile | 58 (3.4) | 45 (4.3) | 0.08 |
| 10$^{th}$-90$^{th}$ centile | 1497 (88.9) | 907 (86.0) | |
| >90$^{th}$ centile | 129 (7.7) | 103 (9.8) | |

Abbreviations: HDP, hypertensive disorder of pregnancy; BMI, body mass index (calculated as weight in kilograms divided by height in meters squared); DIP, diabetes in pregnancy; CNY, Chinese Yuan; ART, assisted reproductive technology.

[a] Statistical significance was tested using an independent unpaired t test for continuous variables, and $\chi^2$ or fisher exact test for categorical variables.

© 2024 Zhu H et al. *JAMA Network Open*.

**eTable 2. The ICD-10 Codes Used to Identify Different Types of Maternal HDP**

| | ICD-10 |
|---|---|
| **Pre-eclampsia** | |
| Moderate pre-eclampsia | O14.0 |
| Severe pre-eclampsia | O14.1 |
| HELLP syndrome | O14.2 |
| Unspecified pre-eclampsia | O14.9 |
| **Eclampsia** | O15 |
| **Gestational hypertension** | |
| Gestational (pregnancy-induced) hypertension | O13 |
| Unspecified maternal hypertension | O16 |
| **Chronic hypertension** | |
| Essential hypertension | I10 |
| Hypertensive heart disease | I11 |
| Hypertensive renal disease | I12 |
| Hypertensive heart and renal disease | I13 |
| Secondary hypertension | I15 |
| Pre-existing hypertension complicating pregnancy | O10 |
| Pre-eclampsia superimposed on chronic hypertension | O11 |

Abbreviations: ICD-10, International Classification of Diseases 10th Revision; HDP, hypertensive disorder of pregnancy.

© 2024 Zhu H et al. *JAMA Network Open*.

## eTable 3. Missing Data for Covariates

| Covariates, No. (%) | Overall (n=3117) |
|---|---|
| Maternal age | 0 (0.0) |
| Maternal prepregnancy BMI | 14 (0.4) |
| Parity | 21 (0.7) |
| Maternal alcohol consumption | 23 (0.7) |
| Maternal smoking | 23 (0.7) |
| Paternal age | 4 (0.1) |
| Paternal hypertension before pregnancy | 0 (0.0) |
| Paternal diabetes before pregnancy | 0 (0.0) |
| Mode of conception | 0 (0.0) |
| Singleton | 0 (0.0) |
| Mode of delivery | 9 (0.3) |
| Gestational week at birth | 12 (0.4) |
| Birth weight | 2 (0.1) |
| Apgar score at 1 minute | 51 (1.6) |
| Apgar score at 5 minutes | 86 (2.8) |
| Offspring sex | 0 (0.0) |
| Offspring congenital abnormality | 12 (0.4) |
| Offspring head circumference | 1433 (46.0) |
| Offspring refractive error | 99 (3.2) |
| Offspring age | 0 (0.0) |
| Household annual income | 112 (3.6) |
| Maternal education level | 10 (0.3) |
| Maternal residence | 1 (0.0) |

Abbreviations: BMI, body mass index (calculated as weight in kilograms divided by height in meters squared).

© 2024 Zhu H et al. *JAMA Network Open*.

**eTable 4. Comparisons of Characteristics Between Offspring With Complete and Incomplete Data of Covariates Adjusted in Model 2[a]**

| Characteristic | With complete data (n=2995) | With incomplete data (n=122) | *P* value[b] |
|---|---|---|---|
| Outcome, No. (%) | | | |
| No | 2644 (88.3) | 105 (86.1) | 0.55 |
| Yes | 351 (11.7) | 17 (13.9) | |
| HDP, No. (%) | | | |
| No | 2854 (95.3) | 120 (98.4) | 0.17 |
| Yes | 141 (4.7) | 2 (1.6) | |
| Maternal age, mean (SD), year | 30.41 (3.76) | 30.16 (3.90) | 0.49 |
| Maternal prepregnancy BMI, No. (%) | | | |
| <24 kg/m$^2$ | 2482 (83.0) | 102 (90.3) | |
| ≥24 kg/m$^2$ | 508 (17.0) | 11 (9.7) | 0.06 |
| Maternal alcohol consumption, No. (%) | | | |
| No | 2928 (98.3) | 115 (99.1) | |
| Yes | 50 (1.7) | 1 (0.9) | 0.76 |
| Maternal smoking, No. (%) | | | |
| No | 2962 (99.5) | 116 (100.0) | 0.90 |
| Yes | 16 (0.5) | 0 (0.0) | |
| Maternal DIP, No. (%) | | | |
| No | 2209 (73.8) | 96 (78.7) | 0.27 |
| Yes | 786 (26.2) | 26 (21.3) | |
| Parity, No. (%) | | | |
| Primipara | 2450 (82.3) | 101 (84.2) | 0.69 |
| Multipara | 526 (17.7) | 19 (15.8) | |
| Maternal education level, No. (%) | | | |
| ≤12 years | 309 (10.3) | 3 (2.7) | 0.01 |
| >12 years | 2686 (89.7) | 109 (97.3) | |
| Maternal residence, No. (%) | | | |
| Country | 482 (16.1) | 10 (8.3) | 0.03 |
| City | 2513 (83.9) | 111 (91.7) | |
| Household annual income, No. (%) | | | |
| <100000 CNY | 1005 (33.6) | 3 (30.0) | |
| 100000-200000 CNY | 1343 (44.8) | 3 (30.0) | 0.35 |
| >200000 CNY | 647 (21.6) | 4 (40.0) | |
| Paternal age, mean (SD), year | 31.92 (4.67) | 31.64 (5.56) | 0.53 |
| Paternal hypertension before pregnancy, No. (%) | | | |
| No | 2985 (99.7) | 122 (100.0) | >.99 |
| Yes | 10 (0.3) | 0 (0.0) | |
| Paternal diabetes before pregnancy, No. (%) | | | |
| No | 2989 (99.8) | 122 (100.0) | >.99 |
| Yes | 6 (0.2) | 0 (0.0) | |
| Mode of conception, No. (%) | | | |

© 2024 Zhu H et al. *JAMA Network Open*.

| | | | |
|---|---|---|---|
| Spontaneously conceived | 2500 (83.5) | 120 (98.4) | <.001 |
| ART | 495 (16.5) | 2 (1.6) | |
| Singleton, No. (%) | | | |
| Yes | 2749 (91.8) | 118 (96.7) | 0.07 |
| No | 246 (8.2) | 4 (3.3) | |
| Mode of delivery, No. (%) | | | |
| Natural delivery | 1595 (53.4) | 80 (65.6) | 0.01 |
| Cesarean section | 1391 (46.6) | 42 (34.4) | |
| Gestational week at birth, mean (SD), week | 38.82 (1.65) | 39.17 (1.34) | 0.03 |
| Birth weight, mean (SD), kilogram | 3.31 (0.49) | 3.42 (0.40) | 0.01 |
| Apgar score at 1 minute, No. (%) | | | |
| >7 | 2933 (99.6) | 122 (100.0) | >.99 |
| ≤7 | 11 (0.4) | 0 (0.0) | |
| Apgar score at 5 minutes, No. (%) | | | |
| >7 | 2910 (100.0) | 121 (100.0) | |
| ≤7 | 0 (0.0) | 0 (0.0) | NA |
| Offspring sex, No. (%) | | | |
| Male | 1554 (51.9) | 75 (61.5) | |
| Female | 1441 (48.1) | 47 (38.5) | 0.05 |
| Offspring congenital abnormality, No. (%) | | | |
| No | 2852 (95.5) | 115 (95.8) | |
| Yes | 133 (4.5) | 5 (4.2) | >.99 |
| Offspring head circumference, No. (%) | | | |
| <10th centile | 58 (3.5) | 0 (0.0) | |
| 10th-90th centile | 1476 (88.9) | 21 (91.3) | 0.65 |
| >90th centile | 127 (7.6) | 2 (8.7) | |
| Offspring refractive error, No. (%) | | | |
| No | 2484 (85.6) | 97 (83.6) | 0.65 |
| Yes | 418 (14.4) | 19 (16.4) | |
| Offspring age, mean (SD), month | 36.29 (0.74) | 36.32 (0.65) | 0.71 |

Abbreviations: HDP, hypertensive disorder of pregnancy; BMI, body mass index (calculated as weight in kilograms divided by height in meters squared); DIP, diabetes in pregnancy;CNY, Chinese Yuan; ART, assisted reproductive technology; NA, not available.

[a] Including maternal age, paternal age, maternal educational level, maternal residence, household annual income, mode of conception, singleton, offspring sex, and offspring age.

[b] Statistical significance was tested using an independent unpaired t test for continuous variables, and $\chi^2$ or fisher exact test for categorical variables.

© 2024 Zhu H et al. *JAMA Network Open*.

## eTable 5. Comparisons of Characteristics Between Offspring With Complete and Incomplete Data of Covariates Adjusted in Model 3[a]

| Characteristic | With complete data (n=2883) | With incomplete data (n=234) | *P* value[b] |
|---|---|---|---|
| Outcome, No. (%) | | | |
| No | 2546 (88.3) | 203 (86.8) | 0.55 |
| Yes | 337 (11.7) | 31 (13.2) | |
| HDP, No. (%) | | | |
| No | 2746 (95.2) | 228 (97.4) | 0.17 |
| Yes | 137 (4.8) | 6 (2.6) | |
| Maternal age, mean (SD), year | 30.43 (3.75) | 30.01 (3.87) | 0.11 |
| Maternal prepregnancy BMI, No. (%) | | | |
| <24 kg/m$^2$ | 2390 (83.0) | 194 (86.2) | |
| ≥24 kg/m$^2$ | 488 (17.0) | 31 (13.8) | 0.26 |
| Maternal alcohol consumption, No. (%) | | | |
| No | 2821 (98.4) | 222 (97.8) | 0.68 |
| Yes | 46 (1.6) | 5 (2.2) | |
| Maternal smoking, No. (%) | | | |
| No | 2851 (99.4) | 227 (100.0) | |
| Yes | 16 (0.6) | 0 (0.0) | 0.52 |
| Maternal DIP, No. (%) | | | |
| No | 2123 (73.6) | 182 (77.8) | |
| Yes | 760 (26.4) | 52 (22.2) | 0.19 |
| Parity, No. (%) | | | |
| Primipara | 2359 (82.3) | 192 (83.1) | |
| Multipara | 506 (17.7) | 39 (16.9) | 0.83 |
| Maternal education level, No. (%) | | | |
| ≤12 years | 298 (10.3) | 14 (6.2) | |
| >12 years | 2585 (89.7) | 210 (93.8) | 0.07 |
| Maternal residence, No. (%) | | | |
| Country | 465 (16.1) | 27 (11.6) | |
| City | 2418 (83.9) | 206 (88.4) | 0.08 |
| Household annual income, No. (%) | | | |
| <100000 CNY | 960 (33.3) | 48 (39.3) | |
| 100000-200000 CNY | 1299 (45.1) | 47 (38.5) | 0.30 |
| >200000 CNY | 624 (21.6) | 27 (22.1) | |
| Paternal age, mean (SD), year | 31.92 (4.65) | 31.67 (5.34) | 0.43 |
| Paternal hypertension before pregnancy, No. (%) | | | |
| No | 2873 (99.7) | 234 (100.0) | 0.76 |
| Yes | 10 (0.3) | 0 (0.0) | |
| Paternal diabetes before pregnancy, No. (%) | | | |
| No | 2877 (99.8) | 234 (100.0) | |
| Yes | 6 (0.2) | 0 (0.0) | >.99 |
| Mode of conception, No. (%) | | | |

© 2024 Zhu H et al. *JAMA Network Open*.

| | | | |
|---|---|---|---|
| Spontaneously conceived | 2401 (83.3) | 219 (93.6) | <.001 |
| ART | 482 (16.7) | 15 (6.4) | |
| Singleton, No. (%) | | | |
| Yes | 2647 (91.8) | 220 (94.0) | 0.29 |
| No | 236 (8.2) | 14 (6.0) | |
| Mode of delivery, No. (%) | | | |
| Natural delivery | 1531 (53.1) | 144 (64.0) | 0.002 |
| Cesarean section | 1352 (46.9) | 81 (36.0) | |
| Gestational week at birth, mean (SD), week | 38.82 (1.64) | 39.11 (1.51) | 0.01 |
| Birth weight, mean (SD), kilogram | 3.31 (0.48) | 3.36 (0.47) | 0.14 |
| Apgar score at 1 minute, No. (%) | | | |
| >7 | 2827 (99.6) | 228 (100.0) | |
| ≤7 | 11 (0.4) | 0 (0.0) | 0.71 |
| Apgar score at 5 minutes, No. (%) | | | |
| >7 | 2804 (100.0) | 227 (100.0) | |
| ≤7 | 0 (0.0) | 0 (0.0) | NA |
| Offspring sex, No. (%) | | | |
| Male | 1506 (52.2) | 123 (52.6) | 0.98 |
| Female | 1377 (47.8) | 111 (47.4) | |
| Offspring congenital abnormality, No. (%) | | | |
| No | 2754 (95.5) | 213 (95.9) | 0.90 |
| Yes | 129 (4.5) | 9 (4.1) | |
| Offspring head circumference, No. (%) | | | |
| <10$^{th}$ centile | 56 (3.5) | 2 (3.2) | |
| 10$^{th}$-90$^{th}$ centile | 1443 (89.0) | 54 (87.1) | 0.83 |
| >90$^{th}$ centile | 123 (7.6) | 6 (9.7) | |
| Offspring refractive error, No. (%) | | | |
| No | 2468 (85.6) | 113 (83.7) | 0.63 |
| Yes | 415 (14.4) | 22 (16.3) | |
| Offspring age, mean (SD), month | 36.30 (0.74) | 36.22 (0.66) | 0.10 |

Abbreviations: HDP, hypertensive disorder of pregnancy; BMI, body mass index (calculated as weight in kilograms divided by height in meters squared); DIP, diabetes in pregnancy;CNY, Chinese Yuan; ART, assisted reproductive technology; NA, not available.

[a] Including maternal age, paternal age, maternal educational level, maternal residence, household annual income, mode of conception, singleton, offspring sex, offspring age, mode of delivery, gestational week at birth, birth weight, and offspring refractive error.

[b] Statistical significance was tested using an independent unpaired t test for continuous variables, and $\chi^2$ or fisher exact test for categorical variables.

© 2024 Zhu H et al. *JAMA Network Open*.

## eTable 6. Relative Risks for the Association Between Maternal DIP and Overall and Specific Types of Strabismus in Offspring

| Outcome, exposure | Outcome | Model 1 | | Model 2 | | Model 3 | |
|---|---|---|---|---|---|---|---|
| | No./total No. (%) | RR (95%CI) | *P* value | RR (95%CI) | *P* value | RR (95%CI) | *P* value |
| **Overall strabismus** | | | | | | | |
| Maternal DIP | | | | | | | |
| No | 272/2305 (11.80) | Reference | | Reference | | Reference | |
| Yes | 96/812 (11.82) | 1(0.79,1.26) | 0.99 | 0.97(0.76,1.24) | 0.82 | 0.95(0.74,1.22) | 0.68 |
| **Exophoria** | | | | | | | |
| Maternal DIP | | | | | | | |
| No | 197/2230 (8.83) | Reference | | Reference | | Reference | |
| Yes | 63/779 (8.09) | 0.92(0.69,1.22) | 0.54 | 0.87(0.64,1.17) | 0.36 | 0.86(0.63,1.17) | 0.34 |
| **Intermittent exotropia** | | | | | | | |
| Maternal DIP | | | | | | | |
| No | 63/2096 (3.01) | Reference | | Reference | | Reference | |
| Yes | 32/748 (4.28) | 1.42(0.93,2.18) | 0.10 | 1.48(0.95,2.3) | 0.08 | 1.33(0.83,2.11) | 0.23 |

Abbreviations: DIP, diabetes in pregnancy; RR, relative risk.

Model 1: Crude. Model 2: Adjusted for maternal age, paternal age, maternal educational level, maternal residence, household annual income, mode of conception, singleton, offspring sex, and offspring age. Model 3: Adjusted for maternal age, paternal age, maternal educational level, maternal residence, household annual income, mode of conception, singleton, offspring sex, offspring age, mode of delivery, gestational week at birth, birth weight, and offspring refractive error.

© 2024 Zhu H et al. *JAMA Network Open*.

eTable 7. Relative Risks for the Association Between Maternal HDP and Overall and Specific Types of Strabismus Restricted to Offspring Without Exposure to Maternal Alcohol Consumption During Pregnancy

| Outcome, exposure | Outcome | Model 1 | | Model 2 | | Model 3 | |
|---|---|---|---|---|---|---|---|
| | No./total No. (%) | RR (95%CI) | *P* value | RR (95%CI) | *P* value | RR (95%CI) | *P* value |
| **All strabismus** | | | | | | | |
| No maternal HDP | 317/2904 (10.92) | Reference | | Reference | | Reference | |
| Maternal HDP | 25/139 (17.99) | 1.65(1.1,2.48) | 0.02 | 1.75(1.14,2.67) | 0.01 | 1.85(1.21,2.84) | 0.005 |
| Hypertension | 12/77 (15.58) | 1.43(0.8,2.54) | 0.23 | 1.46(0.81,2.63) | 0.20 | 1.52(0.84,2.74) | 0.16 |
| Preeclampsia | 13/62 (20.97) | 1.92(1.1,3.34) | 0.02 | 2.15(1.21,3.83) | 0.009 | 2.34(1.32,4.17) | 0.004 |
| Well controlled BP | 4/31 (12.90) | 1.18(0.44,3.17) | 0.74 | 1.22(0.45,3.3) | 0.69 | 1.25(0.46,3.38) | 0.66 |
| Poorly controlled BP | 21/101 (20.79) | 1.9(1.22,2.96) | 0.004 | 2.01(1.27,3.17) | 0.003 | 2.16(1.37,3.42) | 0.001 |
| **Exophoria** | | | | | | | |
| No maternal HDP | 225/2812 (8.00) | Reference | | Reference | | Reference | |
| Maternal HDP | 18/132 (13.64) | 1.7(1.05,2.75) | 0.03 | 1.91(1.16,3.14) | 0.01 | 1.98(1.2,3.26) | 0.008 |
| Hypertension | 7/71 (9.72) | 1.22(0.57,2.58) | 0.61 | 1.28(0.6,2.75) | 0.52 | 1.32(0.61,2.84) | 0.48 |
| Preeclampsia | 11/60 (18.33) | 2.29(1.25,4.2) | 0.007 | 2.81(1.49,5.29) | 0.001 | 2.93(1.55,5.51) | 0.001 |
| Well controlled BP | 3/30 (10.00) | 1.25(0.4,3.9) | 0.70 | 1.36(0.43,4.27) | 0.60 | 1.34(0.43,4.23) | 0.62 |
| Poorly controlled BP | 15/95 (15.79) | 1.97(1.17,3.33) | 0.01 | 2.19(1.28,3.76) | 0.004 | 2.31(1.34,3.97) | 0.002 |
| **Intermittent exotropia** | | | | | | | |
| No maternal HDP | 80/2667 (3.00) | Reference | | Reference | | Reference | |
| Maternal HDP | 7/121 (5.79) | 1.93(0.89,4.17) | 0.10 | 1.76(0.78,3.96) | 0.18 | 2.03(0.89,4.62) | 0.09 |
| Hypertension | 5/70 (7.14) | 2.38(0.97,5.87) | 0.06 | 2.22(0.88,5.63) | 0.09 | 2.43(0.95,6.23) | 0.06 |
| Preeclampsia | 2/51 (3.92) | 1.31(0.32,5.32) | 0.71 | 1.13(0.27,4.78) | 0.87 | 1.41(0.33,6.04) | 0.64 |
| Well controlled BP | 1/28 (3.57) | 1.19(0.17,8.55) | 0.86 | 1.09(0.15,7.88) | 0.93 | 1.27(0.17,9.21) | 0.82 |
| Poorly controlled BP | 6/86 (6.98) | 2.33(1.01,5.33) | 0.05 | 2.09(0.88,5.01) | 0.10 | 2.45(1.01,5.93) | 0.05 |

Abbreviations: HDP, hypertensive disorder of pregnancy; RR, relative risk; BP, blood pressure.

© 2024 Zhu H et al. *JAMA Network Open*.

Model 1: Crude. Model 2: Adjusted for maternal age, paternal age, maternal educational level, maternal residence, household annual income, mode of conception, singleton, offspring sex, and offspring age. Model 3: Adjusted for maternal age, paternal age, maternal educational level, maternal residence, household annual income, mode of conception, singleton, offspring sex, offspring age, mode of delivery, gestational week at birth, birth weight, and offspring refractive error.

© 2024 Zhu H et al. *JAMA Network Open*.

**eTable 8. Relative Risks for the Association Between Maternal HDP and Overall and Specific Types of Strabismus Restricted to Offspring Without Exposure to Maternal Smoking during Pregnancy**

| Outcome, exposure | Outcome No./total No. (%) | Model 1 | | Model 2 | | Model 3 | |
|---|---|---|---|---|---|---|---|
| | | RR (95%CI) | *P* value | RR (95%CI) | *P* value | RR (95%CI) | *P* value |
| **All strabismus** | | | | | | | |
| No maternal HDP | 323/2939 (10.99) | Reference | | Reference | | Reference | |
| Maternal HDP | 24/139 (17.27) | 1.57(1.04,2.38) | 0.03 | 1.67(1.09,2.58) | 0.02 | 1.78(1.16,2.75) | 0.009 |
| Hypertension | 12/78 (15.38) | 1.4(0.79,2.49) | 0.25 | 1.44(0.8,2.59) | 0.22 | 1.51(0.84,2.72) | 0.17 |
| Preeclampsia | 12/61 (19.67) | 1.79(1.01,3.18) | 0.05 | 2.01(1.11,3.66) | 0.02 | 2.19(1.2,3.99) | 0.01 |
| Well controlled BP | 3/31 (9.68) | 0.88(0.28,2.74) | 0.83 | 0.91(0.29,2.87) | 0.88 | 0.94(0.3,2.94) | 0.91 |
| Poorly controlled BP | 21/101 (20.79) | 1.89(1.22,2.94) | 0.005 | 2(1.27,3.16) | 0.003 | 2.16(1.37,3.42) | 0.001 |
| **Exophoria** | | | | | | | |
| No maternal HDP | 228/2844 (8.02) | Reference | | Reference | | Reference | |
| Maternal HDP | 17/132 (12.88) | 1.61(0.98,2.63) | 0.06 | 1.8(1.08,3) | 0.02 | 1.87(1.12,3.13) | 0.02 |
| Hypertension | 7/73 (9.59) | 1.2(0.56,2.54) | 0.64 | 1.26(0.59,2.71) | 0.55 | 1.32(0.61,2.83) | 0.48 |
| Preeclampsia | 10/59 (16.95) | 2.11(1.12,3.98) | 0.02 | 2.59(1.34,5.02) | 0.005 | 2.7(1.39,5.22) | 0.003 |
| Well controlled BP | 2/30 (6.67) | 0.83(0.21,3.35) | 0.80 | 0.9(0.22,3.66) | 0.89 | 0.9(0.22,3.63) | 0.88 |
| Poorly controlled BP | 15/95 (15.79) | 1.97(1.17,3.32) | 0.01 | 2.18(1.27,3.74) | 0.005 | 2.32(1.35,3.99) | 0.002 |
| **Intermittent exotropia** | | | | | | | |
| No maternal HDP | 83/2699 (3.08) | Reference | | Reference | | Reference | |
| Maternal HDP | 7/122 (5.74) | 1.87(0.86,4.03) | 0.11 | 1.73(0.77,3.91) | 0.18 | 2(0.88,4.54) | 0.10 |
| Hypertension | 5/71 (7.04) | 2.29(0.93,5.64) | 0.07 | 2.18(0.86,5.5) | 0.10 | 2.38(0.93,6.09) | 0.07 |
| Preeclampsia | 2/51 (3.92) | 1.28(0.31,5.18) | 0.73 | 1.12(0.26,4.77) | 0.87 | 1.4(0.33,5.99) | 0.65 |
| Well controlled BP | 1/29 (3.45) | 1.12(0.16,8.05) | 0.91 | 1.05(0.14,7.58) | 0.96 | 1.2(0.17,8.73) | 0.86 |
| Poorly controlled BP | 6/86 (6.98) | 2.27(0.99,5.19) | 0.05 | 2.09(0.87,4.98) | 0.10 | 2.45(1.02,5.91) | 0.05 |

Abbreviations: HDP, hypertensive disorder of pregnancy; RR, relative risk; BP, blood pressure.

© 2024 Zhu H et al. *JAMA Network Open*.

Model 1: Crude. Model 2: Adjusted for maternal age, paternal age, maternal educational level, maternal residence, household annual income, mode of conception, singleton, offspring sex, and offspring age. Model 3: Adjusted for maternal age, paternal age, maternal educational level, maternal residence, household annual income, mode of conception, singleton, offspring sex, offspring age, mode of delivery, gestational week at birth, birth weight, and offspring refractive error.

© 2024 Zhu H et al. *JAMA Network Open*.

**eTable 9. Relative Risks for the Association Between Maternal HDP and Overall and Specific Types of Strabismus Restricted to Offspring With Normal Apgar Score**

| Outcome, exposure | Outcome No./total No. (%) | Model 1 | | Model 2 | | Model 3 | |
|---|---|---|---|---|---|---|---|
| | | RR (95%CI) | *P* value | RR (95%CI) | *P* value | RR (95%CI) | *P* value |
| **All strabismus** | | | | | | | |
| No maternal HDP | 334/2884 (11.58) | Reference | | Reference | | Reference | |
| Maternal HDP | 28/137 (20.44) | 1.76(1.2,2.6) | 0.004 | 1.74(1.16,2.61) | 0.008 | 1.86(1.24,2.8) | 0.003 |
| Hypertension | 12/72 (16.67) | 1.44(0.81,2.56) | 0.22 | 1.43(0.8,2.56) | 0.23 | 1.5(0.83,2.69) | 0.18 |
| Preeclampsia | 16/65 (24.62) | 2.13(1.29,3.51) | 0.003 | 2.12(1.24,3.65) | 0.006 | 2.33(1.36,4.01) | 0.002 |
| Well controlled BP | 5/33 (15.15) | 1.31(0.54,3.16) | 0.55 | 1.33(0.54,3.23) | 0.53 | 1.37(0.56,3.34) | 0.49 |
| Poorly controlled BP | 23/103 (22.33) | 1.93(1.26,2.94) | 0.002 | 1.89(1.21,2.95) | 0.005 | 2.05(1.31,3.2) | 0.002 |
| **Exophoria** | | | | | | | |
| No maternal HDP | 235/2785 (8.44) | Reference | | Reference | | Reference | |
| Maternal HDP | 19/128 (14.84) | 1.76(1.1,2.81) | 0.02 | 1.81(1.1,2.97) | 0.02 | 1.9(1.16,3.12) | 0.01 |
| Hypertension | 7/67 (10.45) | 1.24(0.58,2.63) | 0.58 | 1.26(0.59,2.7) | 0.55 | 1.32(0.61,2.83) | 0.48 |
| Preeclampsia | 12/61 (19.67) | 2.33(1.31,4.16) | 0.004 | 2.54(1.35,4.78) | 0.004 | 2.68(1.42,5.05) | 0.002 |
| Well controlled BP | 3/31 (9.68) | 1.15(0.37,3.58) | 0.81 | 1.25(0.4,3.94) | 0.70 | 1.25(0.4,3.95) | 0.70 |
| Poorly controlled BP | 16/96 (16.67) | 1.98(1.19,3.28) | 0.008 | 2.01(1.17,3.44) | 0.01 | 2.14(1.25,3.68) | 0.006 |
| **Intermittent exotropia** | | | | | | | |
| No maternal HDP | 86/2636 (3.26) | Reference | | Reference | | Reference | |
| Maternal HDP | 9/118 (7.63) | 2.34(1.18,4.64) | 0.02 | 2.02(0.98,4.16) | 0.06 | 2.35(1.13,4.89) | 0.02 |
| Hypertension | 5/65 (7.69) | 2.36(0.96,5.81) | 0.06 | 2.17(0.86,5.45) | 0.10 | 2.41(0.95,6.11) | 0.07 |
| Preeclampsia | 4/53 (7.55) | 2.31(0.85,6.3) | 0.10 | 1.84(0.64,5.28) | 0.26 | 2.28(0.79,6.61) | 0.13 |
| Well controlled BP | 2/30 (6.67) | 2.04(0.5,8.3) | 0.32 | 1.82(0.44,7.47) | 0.41 | 2.13(0.52,8.8) | 0.30 |
| Poorly controlled BP | 7/87 (8.05) | 2.47(1.14,5.33) | 0.02 | 2.1(0.93,4.71) | 0.07 | 2.45(1.07,5.57) | 0.03 |

Abbreviations: HDP, hypertensive disorder of pregnancy; RR, relative risk; BP, blood pressure.

© 2024 Zhu H et al. *JAMA Network Open*.

Model 1: Crude. Model 2: Adjusted for maternal age, paternal age, maternal educational level, maternal residence, household annual income, mode of conception, singleton, offspring sex, and offspring age. Model 3: Adjusted for maternal age, paternal age, maternal educational level, maternal residence, household annual income, mode of conception, singleton, offspring sex, offspring age, mode of delivery, gestational week at birth, birth weight, and offspring refractive error.

© 2024 Zhu H et al. *JAMA Network Open*.

**eTable 10. Relative Risks for the Association Between Maternal HDP and Overall and Specific Types of Strabismus Restricted to Offspring Without Congenital Abnormality**

| Outcome, exposure | Outcome No./total No. (%) | Model 1 | | Model 2 | | Model 3 | |
|---|---|---|---|---|---|---|---|
| | | RR (95%CI) | *P* value | RR (95%CI) | *P* value | RR (95%CI) | *P* value |
| **All strabismus** | | | | | | | |
| No maternal HDP | 323/2834 (11.40) | Reference | | Reference | | Reference | |
| Maternal HDP | 27/133 (20.30) | 1.78(1.2,2.64) | 0.004 | 1.74(1.15,2.63) | 0.009 | 1.83(1.2,2.77) | 0.005 |
| Hypertension | 11/74 (14.86) | 1.3(0.72,2.38) | 0.39 | 1.32(0.72,2.42) | 0.38 | 1.37(0.75,2.53) | 0.31 |
| Preeclampsia | 16/59 (27.12) | 2.38(1.44,3.93) | 0.001 | 2.3(1.34,3.96) | 0.002 | 2.43(1.42,4.19) | 0.001 |
| Well controlled BP | 4/31 (12.90) | 1.13(0.42,3.03) | 0.81 | 1.14(0.42,3.06) | 0.80 | 1.17(0.43,3.16) | 0.75 |
| Poorly controlled BP | 23/97 (23.71) | 2.08(1.36,3.18) | 0.001 | 2.01(1.28,3.14) | 0.002 | 2.12(1.36,3.32) | 0.001 |
| **Exophoria** | | | | | | | |
| No maternal HDP | 230/2741 (8.39) | Reference | | Reference | | Reference | |
| Maternal HDP | 18/124 (14.52) | 1.73(1.07,2.79) | 0.03 | 1.74(1.04,2.89) | 0.03 | 1.79(1.07,2.98) | 0.03 |
| Hypertension | 6/69 (8.70) | 1.04(0.46,2.33) | 0.93 | 1.07(0.47,2.43) | 0.87 | 1.11(0.49,2.53) | 0.80 |
| Preeclampsia | 12/55 (21.82) | 2.6(1.46,4.65) | 0.001 | 2.68(1.42,5.04) | 0.002 | 2.72(1.44,5.12) | 0.002 |
| Well controlled BP | 2/29 (6.90) | 0.82(0.2,3.31) | 0.78 | 0.88(0.22,3.55) | 0.85 | 0.87(0.22,3.54) | 0.85 |
| Poorly controlled BP | 16/90 (17.78) | 2.12(1.28,3.52) | 0.004 | 2.09(1.22,3.58) | 0.007 | 2.17(1.27,3.73) | 0.005 |
| **Intermittent exotropia** | | | | | | | |
| No maternal HDP | 82/2593 (3.16) | Reference | | Reference | | Reference | |
| Maternal HDP | 9/115 (7.83) | 2.47(1.24,4.92) | 0.01 | 2.2(1.06,4.57) | 0.03 | 2.57(1.23,5.38) | 0.01 |
| Hypertension | 5/68 (7.35) | 2.33(0.94,5.73) | 0.07 | 2.21(0.88,5.59) | 0.09 | 2.5(0.98,6.38) | 0.06 |
| Preeclampsia | 4/47 (8.51) | 2.69(0.99,7.34) | 0.05 | 2.19(0.76,6.29) | 0.15 | 2.67(0.92,7.74) | 0.07 |
| Well controlled BP | 2/29 (6.90) | 2.18(0.54,8.86) | 0.28 | 1.98(0.48,8.18) | 0.34 | 2.34(0.56,9.68) | 0.24 |
| Poorly controlled BP | 7/81 (8.64) | 2.73(1.26,5.91) | 0.01 | 2.41(1.07,5.42) | 0.03 | 2.82(1.24,6.4) | 0.01 |

Abbreviations: HDP, hypertensive disorder of pregnancy; RR, relative risk; BP, blood pressure.

© 2024 Zhu H et al. *JAMA Network Open*.

Model 1: Crude. Model 2: Adjusted for maternal age, paternal age, maternal educational level, maternal residence, household annual income, mode of conception, singleton, offspring sex, and offspring age. Model 3: Adjusted for maternal age, paternal age, maternal educational level, maternal residence, household annual income, mode of conception, singleton, offspring sex, offspring age, mode of delivery, gestational week at birth, birth weight, and offspring refractive error.

© 2024 Zhu H et al. *JAMA Network Open*.

**eTable 11. Relative Risks for the Association Between Maternal HDP and Overall and Specific Types of Strabismus Restricted to Offspring With Normal Head Circumference**

| Outcome, exposure | Outcome No./total No. (%) | Model 1 | | Model 2 | | Model 3 | |
|---|---|---|---|---|---|---|---|
| | | RR (95%CI) | *P* value | RR (95%CI) | *P* value | RR (95%CI) | *P* value |
| **All strabismus** | | | | | | | |
| No maternal HDP | 153/1442 (10.61) | Reference | | Reference | | Reference | |
| Maternal HDP | 12/55 (21.82) | 2.06(1.14,3.7) | 0.01 | 1.83(0.98,3.43) | 0.06 | 1.96(1.04,3.71) | 0.04 |
| Hypertension | 5/30 (16.67) | 1.57(0.64,3.83) | 0.32 | 1.42(0.58,3.51) | 0.45 | 1.53(0.61,3.8) | 0.36 |
| Preeclampsia | 7/25 (28.00) | 2.64(1.24,5.63) | 0.01 | 2.4(1.04,5.55) | 0.04 | 2.57(1.11,5.96) | 0.03 |
| Well controlled BP | 1/14 (7.14) | 0.67(0.09,4.81) | 0.69 | 0.58(0.08,4.17) | 0.56 | 0.62(0.08,4.47) | 0.63 |
| Poorly controlled BP | 11/41 (26.83) | 2.53(1.37,4.66) | 0.003 | 2.33(1.21,4.49) | 0.01 | 2.51(1.29,4.87) | 0.007 |
| **Exophoria** | | | | | | | |
| No maternal HDP | 104/1393 (7.47) | Reference | | Reference | | Reference | |
| Maternal HDP | 9/52 (17.31) | 2.32(1.17,4.58) | 0.02 | 2.1(1,4.4) | 0.05 | 2.21(1.05,4.68) | 0.04 |
| Hypertension | 3/28 (10.71) | 1.44(0.46,4.52) | 0.54 | 1.29(0.4,4.14) | 0.67 | 1.37(0.42,4.42) | 0.60 |
| Preeclampsia | 6/24 (25.00) | 3.35(1.47,7.63) | 0.004 | 3.35(1.34,8.36) | 0.01 | 3.47(1.38,8.74) | 0.008 |
| Well controlled BP | 1/14 (7.14) | 0.96(0.13,6.86) | 0.97 | 0.84(0.11,6.12) | 0.86 | 0.88(0.12,6.43) | 0.90 |
| Poorly controlled BP | 8/38 (21.05) | 2.82(1.37,5.79) | 0.005 | 2.66(1.22,5.83) | 0.01 | 2.81(1.27,6.21) | 0.01 |
| **Intermittent exotropia** | | | | | | | |
| No maternal HDP | 47/1336 (3.52) | Reference | | Reference | | Reference | |
| Maternal HDP[a] | 3/46 (6.52) | 1.85(0.58,5.95) | 0.30 | 1.54(0.46,5.18) | 0.49 | 1.71(0.49,5.89) | 0.40 |
| Hypertension | 2/27 (7.41) | 2.11(0.51,8.66) | 0.30 | 1.9(0.45,8) | 0.38 | 2.21(0.51,9.47) | 0.29 |
| Preeclampsia | 1/19 (5.26) | 1.5(0.21,10.84) | 0.69 | 1.08(0.14,8.57) | 0.94 | 1.13(0.14,9.25) | 0.91 |

Abbreviations: HDP, hypertensive disorder of pregnancy; RR, Relative Risk; BP, blood pressure.

Model 1: Crude. Model 2: Adjusted for maternal age, paternal age, maternal educational level, maternal residence, household annual income, mode of conception, singleton, offspring sex, and offspring age. Model 3: Adjusted for maternal age, paternal age, maternal educational level, maternal residence, household annual income, mode of conception, singleton, offspring sex, offspring age, mode of

© 2024 Zhu H et al. *JAMA Network Open*.

delivery, gestational week at birth, birth weight, and offspring refractive error.

[a] Maternal HDP was not categorized into with BP well controlled and poorly controlled due to small number.

© 2024 Zhu H et al. *JAMA Network Open*.

**eTable 12. Relative Risks for the Association Between Maternal HDP and Overall and Specific Types of Strabismus Restricted to Offspring Born to Primipara**

| Outcome, exposure | Outcome No./total No. (%) | Model 1 | | Model 2 | | Model 3 | |
|---|---|---|---|---|---|---|---|
| | | RR (95%CI) | *P* value | RR (95%CI) | *P* value | RR (95%CI) | *P* value |
| **All strabismus** | | | | | | | |
| No maternal HDP | 267/2433 (10.97) | Reference | | Reference | | Reference | |
| Maternal HDP | 21/118 (17.80) | 1.62(1.04,2.53) | 0.03 | 1.8(1.13,2.86) | 0.01 | 1.88(1.18,3) | 0.008 |
| Hypertension | 9/64 (14.06) | 1.28(0.66,2.49) | 0.46 | 1.41(0.72,2.76) | 0.32 | 1.43(0.73,2.82) | 0.30 |
| Preeclampsia | 12/54 (22.22) | 2.02(1.14,3.61) | 0.02 | 2.3(1.26,4.2) | 0.006 | 2.48(1.36,4.53) | 0.003 |
| Well controlled BP | 4/27 (14.81) | 1.35(0.5,3.62) | 0.55 | 1.5(0.56,4.07) | 0.42 | 1.52(0.56,4.1) | 0.41 |
| Poorly controlled BP | 17/84 (20.24) | 1.84(1.13,3.01) | 0.01 | 2.01(1.21,3.33) | 0.007 | 2.13(1.28,3.55) | 0.004 |
| **Exophoria** | | | | | | | |
| No maternal HDP | 188/2354 (7.99) | Reference | | Reference | | Reference | |
| Maternal HDP | 14/111 (12.61) | 1.58(0.92,2.72) | 0.10 | 1.83(1.04,3.21) | 0.04 | 1.88(1.07,3.32) | 0.03 |
| Hypertension | 4/59 (6.78) | 0.85(0.32,2.29) | 0.75 | 0.95(0.35,2.58) | 0.92 | 0.97(0.36,2.65) | 0.96 |
| Preeclampsia | 10/52 (19.23) | 2.41(1.27,4.55) | 0.007 | 2.96(1.52,5.74) | 0.001 | 3.05(1.57,5.93) | 0.001 |
| Well controlled BP | 3/26 (11.54) | 1.44(0.46,4.52) | 0.53 | 1.67(0.53,5.26) | 0.38 | 1.64(0.52,5.19) | 0.40 |
| Poorly controlled BP | 11/78 (14.10) | 1.77(0.96,3.24) | 0.07 | 2(1.07,3.74) | 0.03 | 2.09(1.11,3.93) | 0.02 |
| **Intermittent exotropia** | | | | | | | |
| No maternal HDP | 68/2234 (3.04) | Reference | | Reference | | Reference | |
| Maternal HDP | 7/104 (6.73) | 2.21(1.02,4.81) | 0.05 | 2.23(0.98,5.1) | 0.06 | 2.54(1.09,5.88) | 0.03 |
| Hypertension | 5/60 (8.33) | 2.74(1.1,6.79) | 0.03 | 2.89(1.13,7.39) | 0.03 | 3.03(1.16,7.92) | 0.02 |
| Preeclampsia | 2/44 (4.55) | 1.49(0.37,6.09) | 0.58 | 1.39(0.32,5.94) | 0.66 | 1.78(0.41,7.69) | 0.44 |
| Well controlled BP | 1/24 (4.17) | 1.37(0.19,9.85) | 0.76 | 1.36(0.19,9.91) | 0.76 | 1.56(0.21,11.43) | 0.66 |
| Poorly controlled BP | 6/73 (8.22) | 2.7(1.17,6.22) | 0.02 | 2.71(1.12,6.55) | 0.03 | 3.14(1.28,7.75) | 0.01 |

Abbreviations: HDP, hypertensive disorder of pregnancy; RR, relative risk; BP, blood pressure.

© 2024 Zhu H et al. *JAMA Network Open*.

Model 1: Crude. Model 2: Adjusted for maternal age, paternal age, maternal educational level, maternal residence, household annual income, mode of conception, singleton, offspring sex, and offspring age. Model 3: Adjusted for maternal age, paternal age, maternal educational level, maternal residence, household annual income, mode of conception, singleton, offspring sex, offspring age, mode of delivery, gestational week at birth, birth weight, and offspring refractive error.

© 2024 Zhu H et al. *JAMA Network Open*.

**eTable 13. Relative Risks for the Association Between Maternal HDP and Overall and Specific Types of Strabismus Restricted to Term-born Children of Normal Birth Weight**

| Outcome, exposure | Outcome No./total No. (%) | Model 1 | | Model 2 | | Model 3 | |
|---|---|---|---|---|---|---|---|
| | | RR (95%CI) | *P* value | RR (95%CI) | *P* value | RR (95%CI) | *P* value |
| **All strabismus** | | | | | | | |
| No maternal HDP | 318/2729 (11.65) | Reference | | Reference | | Reference | |
| Maternal HDP | 23/97 (23.71) | 2.03(1.33,3.11) | 0.001 | 1.88(1.21,2.93) | 0.005 | 1.97(1.26,3.08) | 0.003 |
| Hypertension | 11/61 (18.03) | 1.55(0.85,2.82) | 0.15 | 1.46(0.79,2.69) | 0.23 | 1.52(0.82,2.81) | 0.18 |
| Preeclampsia | 12/36 (33.33) | 2.86(1.61,5.09) | <.001 | 2.61(1.42,4.81) | 0.002 | 2.78(1.51,5.12) | 0.001 |
| Well controlled BP | 3/28 (10.71) | 0.92(0.29,2.87) | 0.89 | 0.88(0.28,2.78) | 0.83 | 0.91(0.29,2.88) | 0.88 |
| Poorly controlled BP | 20/68 (29.41) | 2.52(1.61,3.97) | <.001 | 2.3(1.43,3.68) | 0.001 | 2.43(1.52,3.91) | <.001 |
| **Exophoria** | | | | | | | |
| No maternal HDP | 226/2637 (8.57) | Reference | | Reference | | Reference | |
| Maternal HDP | 16/90 (17.78) | 2.07(1.25,3.44) | 0.005 | 2.01(1.18,3.42) | 0.01 | 2.06(1.21,3.51) | 0.008 |
| Hypertension | 7/57 (12.28) | 1.43(0.68,3.04) | 0.35 | 1.41(0.66,3.02) | 0.38 | 1.46(0.68,3.13) | 0.33 |
| Preeclampsia | 9/33 (27.27) | 3.18(1.63,6.2) | 0.001 | 3.15(1.54,6.43) | 0.002 | 3.17(1.55,6.49) | 0.002 |
| Well controlled BP | 2/27 (7.41) | 0.86(0.21,3.48) | 0.84 | 0.93(0.23,3.78) | 0.92 | 0.93(0.23,3.76) | 0.91 |
| Poorly controlled BP | 14/62 (22.58) | 2.63(1.54,4.52) | <.001 | 2.46(1.4,4.34) | 0.002 | 2.56(1.45,4.52) | 0.001 |
| **Intermittent exotropia** | | | | | | | |
| No maternal HDP | 79/2490 (3.17) | Reference | | Reference | | Reference | |
| Maternal HDP | 7/81 (8.64) | 2.72(1.26,5.9) | 0.01 | 2.16(0.97,4.84) | 0.06 | 2.44(1.08,5.49) | 0.03 |
| Hypertension | 4/54 (7.41) | 2.33(0.86,6.37) | 0.10 | 1.9(0.67,5.35) | 0.23 | 1.98(0.69,5.64) | 0.20 |
| Preeclampsia | 3/27 (11.11) | 3.5(1.11,11.08) | 0.03 | 2.65(0.82,8.61) | 0.11 | 3.45(1.06,11.2) | 0.04 |
| Well controlled BP | 126 (3.85) | 1.21(0.17,8.71) | 0.85 | 0.95(0.13,6.91) | 0.96 | 1.07(0.15,7.85) | 0.95 |
| Poorly controlled BP | 6/54 (11.11) | 3.5(1.53,8.03) | 0.003 | 2.77(1.17,6.56) | 0.02 | 3.14(1.32,7.49) | 0.01 |

Abbreviations: HDP, hypertensive disorder of pregnancy; RR, relative risk; BP, blood pressure.

© 2024 Zhu H et al. *JAMA Network Open*.

Model 1: Crude. Model 2: Adjusted for maternal age, paternal age, maternal educational level, maternal residence, household annual income, mode of conception, singleton, offspring sex, and offspring age. Model 3: Adjusted for maternal age, paternal age, maternal educational level, maternal residence, household annual income, mode of conception, singleton, offspring sex, offspring age, mode of delivery, gestational week at birth, birth weight, and offspring refractive error.

© 2024 Zhu H et al. *JAMA Network Open*.

**eTable 14. Relative Risks for the Association Between Maternal HDP and Overall and Specific Types of Strabismus after Excluding Children With Exposure to Paternal Hypertension or Diabetes before Pregnancy**

| Outcome, exposure | Outcome No./total No. (%) | Model 1 | | Model 2 | | Model 3 | |
|---|---|---|---|---|---|---|---|
| | | RR (95%CI) | *P* value | RR (95%CI) | *P* value | RR (95%CI) | *P* value |
| **All strabismus** | | | | | | | |
| No maternal HDP | 336/2958 (11.36) | Reference | | Reference | | Reference | |
| Maternal HDP | 28/143 (19.58) | 1.72(1.17,2.53) | 0.006 | 1.71(1.14,2.58) | 0.01 | 1.83(1.21,2.76) | 0.004 |
| Hypertension | 12/124 (9.68) | 1.35(0.76,2.41) | 0.30 | 1.36(0.76,2.45) | 0.30 | 1.42(0.79,2.56) | 0.24 |
| Preeclampsia | 16/65 (24.62) | 2.17(1.31,3.58) | 0.003 | 2.18(1.27,3.74) | 0.005 | 2.4(1.4,4.11) | 0.001 |
| Well controlled BP | 5/33 (15.15) | 1.33(0.55,3.23) | 0.52 | 1.36(0.56,3.32) | 0.49 | 1.41(0.58,3.43) | 0.45 |
| Poorly controlled BP | 23/103 (22.33) | 1.97(1.29,3) | 0.002 | 1.93(1.23,3.01) | 0.004 | 2.09(1.33,3.26) | 0.001 |
| **Exophoria** | | | | | | | |
| No maternal HDP | 238/2860 (8.32) | Reference | | Reference | | Reference | |
| Maternal HDP | 19/134 (14.18) | 1.7(1.07,2.72) | 0.03 | 1.75(1.07,2.88) | 0.03 | 1.84(1.11,3.02) | 0.02 |
| Hypertension | 7/73 (9.59) | 1.15(0.54,2.44) | 0.71 | 1.19(0.56,2.54) | 0.66 | 1.23(0.57,2.64) | 0.59 |
| Preeclampsia | 12/61 (19.67) | 2.36(1.32,4.22) | 0.004 | 2.55(1.36,4.8) | 0.004 | 2.7(1.44,5.07) | 0.002 |
| Well controlled BP | 3/31 (9.68) | 1.16(0.37,3.63) | 0.80 | 1.26(0.4,3.98) | 0.69 | 1.26(0.4,3.96) | 0.70 |
| Poorly controlled BP | 16/96 (16.67) | 2(1.21,3.32) | 0.007 | 2.01(1.18,3.45) | 0.01 | 2.14(1.25,3.68) | 0.006 |
| **Intermittent exotropia** | | | | | | | |
| No maternal HDP | 85/2707 (3.14) | Reference | | Reference | | Reference | |
| Maternal HDP | 9/124 (7.26) | 2.31(1.16,4.59) | 0.02 | 2.06(0.99,4.26) | 0.05 | 2.39(1.14,4.99) | 0.02 |
| Hypertension | 5/71 (7.04) | 2.24(0.91,5.52) | 0.08 | 2.1(0.84,5.3) | 0.11 | 2.3(0.9,5.85) | 0.08 |
| Preeclampsia | 4/53 (7.55) | 2.4(0.88,6.55) | 0.09 | 2(0.7,5.72) | 0.20 | 2.52(0.87,7.29) | 0.09 |
| Well controlled BP | 2/30 (6.67) | 2.12(0.52,8.62) | 0.29 | 1.94(0.47,7.97) | 0.36 | 2.28(0.55,9.42) | 0.25 |
| Poorly controlled BP | 7/87 (8.05) | 2.56(1.19,5.54) | 0.02 | 2.25(1,5.05) | 0.05 | 2.65(1.17,6.02) | 0.02 |

Abbreviations: HDP, hypertensive disorder of pregnancy; RR, relative risk; BP, blood pressure.

© 2024 Zhu H et al. *JAMA Network Open*.

Model 1: Crude. Model 2: Adjusted for maternal age, paternal age, maternal educational level, maternal residence, household annual income, mode of conception, singleton, offspring sex, and offspring age. Model 3: Adjusted for maternal age, paternal age, maternal educational level, maternal residence, household annual income, mode of conception, singleton, offspring sex, offspring age, mode of delivery, gestational week at birth, birth weight, and offspring refractive error.

© 2024 Zhu H et al. *JAMA Network Open*.

**eTable 15. Relative Risks for the Association Between Maternal HDP and Overall and Specific Types of Strabismus Restricted to Children Born to Mothers With Prepregnancy BMI <24**

| Outcome, exposure | Outcome No./total No. (%) | Model 1 | | Model 2 | | Model 3 | |
|---|---|---|---|---|---|---|---|
| | | RR (95%CI) | *P* value | RR (95%CI) | *P* value | RR (95%CI) | *P* value |
| **All strabismus** | | | | | | | |
| No maternal HDP | 284/2500 (11.36) | Reference | | Reference | | Reference | Reference |
| Maternal HDP | 17/84 (20.24) | 1.78(1.09,2.91) | 0.02 | 1.85(1.11,3.09) | 0.02 | 2.02(1.21,3.37) | 0.007 |
| Hypertension | 4/35 (11.43) | 1.01(0.37,2.7) | 0.99 | 1.01(0.37,2.72) | 0.99 | 1.07(0.39,2.89) | 0.90 |
| Preeclampsia | 13/49 (26.53) | 2.34(1.34,4.07) | 0.003 | 2.54(1.41,4.56) | 0.002 | 2.83(1.58,5.07) | <0.001 |
| Well controlled BP | 2/21 (9.52) | 0.84(0.21,3.37) | 0.80 | 0.85(0.21,3.41) | 0.81 | 0.86(0.21,3.46) | 0.83 |
| Poorly controlled BP | 15/61 (24.59) | 2.16(1.29,3.64) | 0.004 | 2.28(1.32,3.93) | 0.003 | 2.56(1.48,4.41) | 0.001 |
| **Exophoria** | | | | | | | |
| No maternal HDP | 204/2420 (8.43) | Reference | | Reference | | Reference | |
| Maternal HDP | 10/77 (12.99) | 1.54(0.82,2.91) | 0.18 | 1.69(0.87,3.26) | 0.12 | 1.78(0.92,3.44) | 0.09 |
| Hypertension | 1/32 (3.13) | 0.37(0.05,2.64) | 0.32 | 0.38(0.05,2.75) | 0.34 | 0.4(0.06,2.88) | 0.36 |
| Preeclampsia | 9/45 (20.00) | 2.37(1.22,4.63) | 0.01 | 2.78(1.38,5.61) | 0.004 | 2.94(1.46,5.92) | 0.003 |
| Well controlled BP | 1/20 (5.00) | 0.59(0.08,4.23) | 0.60 | 0.63(0.09,4.54) | 0.65 | 0.61(0.08,4.37) | 0.62 |
| Poorly controlled BP | 9/55 (16.36) | 1.94(1,3.78) | 0.05 | 2.15(1.07,4.3) | 0.03 | 2.34(1.17,4.69) | 0.02 |
| **Intermittent exotropia** | | | | | | | |
| No maternal HDP | 69/2285 (3.02) | Reference | | Reference | | Reference | |
| Maternal HDP | 7/74 (9.46) | 3.13(1.44,6.81) | 0.004 | 2.95(1.28,6.79) | 0.01 | 3.9(1.69,9.03) | 0.001 |
| Hypertension | 3/34 (8.82) | 2.92(0.92,9.28) | 0.07 | 2.85(0.88,9.26) | 0.08 | 3.42(1.04,11.2) | 0.04 |
| Preeclampsia | 4/40 (10.00) | 3.31(1.21,9.07) | 0.02 | 3.04(1.03,8.94) | 0.04 | 4.41(1.5,12.97) | 0.007 |
| Well controlled BP | 1/20 (5.00) | 1.66(0.23,11.91) | 0.62 | 1.56(0.21,11.38) | 0.66 | 1.93(0.26,14.12) | 0.52 |
| Poorly controlled BP | 6/52 (11.54) | 3.82(1.66,8.79) | 0.002 | 3.63(1.47,8.99) | 0.005 | 4.99(2.01,12.41) | 0.001 |

Abbreviations: HDP, hypertensive disorder of pregnancy; RR, Relative Risk; BP, blood pressure; BMI, body mass index (calculated as weight in kilograms divided by height in meters squared).

© 2024 Zhu H et al. *JAMA Network Open*.

Model 1: Crude. Model 2: Adjusted for maternal age, paternal age, maternal educational level, maternal residence, household annual income, mode of conception, singleton, offspring sex, and offspring age. Model 3: Adjusted for maternal age, paternal age, maternal educational level, maternal residence, household annual income, mode of conception, singleton, offspring sex, offspring age, mode of delivery, gestational week at birth, birth weight, and offspring refractive error.

© 2024 Zhu H et al. *JAMA Network Open*.

**eTable 16. Relative Risks for the Association Between Maternal HDP and Overall and Specific Types of Strabismus after PSM**

| Outcome, exposure | Outcome No./total No. (%) | Model 1 | | Model 2 | | Model 3 | |
|---|---|---|---|---|---|---|---|
| | | RR (95%CI) | *P* value | RR (95%CI) | *P* value | RR (95%CI) | *P* value |
| **All strabismus** | | | | | | | |
| No maternal HDP | 60/540 (11.11) | Reference | | Reference | | Reference | |
| Maternal HDP | 27/137 (19.71) | 1.77(1.13,2.79) | 0.013 | 1.76(1.11,2.77) | 0.02 | 1.79(1.13,2.84) | 0.01 |
| Hypertension | 12/77 (15.58) | 1.4(0.75,2.61) | 0.285 | 1.38(0.74,2.59) | 0.31 | 1.4(0.75,2.63) | 0.30 |
| Preeclampsia | 1560 (25.00) | 2.25(1.28,3.96) | 0.005 | 2.24(1.25,4.02) | 0.006 | 2.33(1.29,4.18) | 0.005 |
| Well controlled BP | 5/33 (15.15) | 1.36(0.55,3.4) | 0.505 | 1.43(0.57,3.58) | 0.45 | 1.43(0.57,3.6) | 0.45 |
| Poorly controlled BP | 22/97 (22.68) | 2.04(1.25,3.33) | 0.004 | 1.97(1.2,3.22) | 0.007 | 2.03(1.24,3.32) | 0.005 |
| **Exophoria** | | | | | | | |
| No maternal HDP | 42/522 (8.05) | Reference | | Reference | | Reference | |
| Maternal HDP | 18/128 (14.06) | 1.75(1.01,3.04) | 0.047 | 1.74(1,3.03) | 0.05 | 1.75(1,3.05) | 0.05 |
| Hypertension | 7/72 (9.72) | 1.21(0.54,2.69) | 0.643 | 1.17(0.52,2.63) | 0.70 | 1.18(0.52,2.65) | 0.69 |
| Preeclampsia | 11/56 (19.64) | 2.44(1.26,4.74) | 0.008 | 2.52(1.27,5) | 0.008 | 2.53(1.27,5.05) | 0.008 |
| Well controlled BP | 3/31 (9.68) | 1.2(0.37,3.88) | 0.757 | 1.31(0.4,4.29) | 0.65 | 1.28(0.39,4.19) | 0.68 |
| Poorly controlled BP | 15/90 (16.67) | 2.07(1.15,3.74) | 0.015 | 1.99(1.1,3.61) | 0.02 | 2.01(1.11,3.65) | 0.02 |
| **Intermittent exotropia** | | | | | | | |
| No maternal HDP | 16/496 (3.23) | Reference | | Reference | | Reference | |
| Maternal HDP | 9/119 (7.56) | 2.34(1.04,5.3) | 0.041 | 2.24(0.98,5.13) | 0.06 | 2.56(1.1,5.93) | 0.03 |
| Hypertension | 5/70 (7.14) | 2.21(0.81,6.04) | 0.121 | 2.3(0.83,6.36) | 0.11 | 2.7(0.95,7.66) | 0.06 |
| Preeclampsia | 4/49 (8.16) | 2.53(0.85,7.57) | 0.097 | 2.18(0.7,6.78) | 0.18 | 2.39(0.76,7.56) | 0.14 |
| Well controlled BP | 2/30 (6.67) | 2.07(0.48,8.99) | 0.333 | 1.9(0.43,8.43) | 0.40 | 2.05(0.45,9.36) | 0.36 |
| Poorly controlled BP | 7/82 (8.54) | 2.65(1.09,6.43) | 0.032 | 2.53(1.03,6.22) | 0.04 | 2.99(1.2,7.45) | 0.02 |

Abbreviations: HDP, hypertensive disorder of pregnancy; RR, relative risk; BP, blood pressure; PSM, propensity score matching.

© 2024 Zhu H et al. *JAMA Network Open*.

Model 1: Crude. Model 2: Adjusted for maternal age, paternal age, maternal educational level, maternal residence, household annual income, mode of conception, singleton, offspring sex, and offspring age. Model 3: Adjusted for maternal age, paternal age, maternal educational level, maternal residence, household annual income, mode of conception, singleton, offspring sex, offspring age, mode of delivery, gestational week at birth, birth weight, and offspring refractive error.

© 2024 Zhu H et al. *JAMA Network Open*.

**eTable 17. Direct Comparisons of Overall Strabismus Risk in Offspring Between Different HDP Types, BP Control Levels, or Their Combinations**

| Exposure | Outcome No./total No. (%) | Model 1 | | Model 2 | | Model 3 | |
|---|---|---|---|---|---|---|---|
| | | RR (95%CI) | *P* value | RR (95%CI) | *P* value | RR (95%CI) | *P* value |
| **Type of maternal HDP** | | | | | | | |
| Hypertension | 12/78 (15.38) | Reference | | Reference | | Reference | |
| Preeclampsia | 16/65 (24.62) | 1.6(0.76,3.38) | 0.22 | 1.64(0.7,3.82) | 0.26 | 1.64(0.68,3.92) | 0.27 |
| **BP control level of maternal HDP** | | | | | | | |
| Well controlled BP | 5/33 (15.15) | Reference | | Reference | | Reference | |
| Poorly controlled BP | 23/103 (22.33) | 1.47(0.56,3.88) | 0.43 | 1.26(0.46,3.43) | 0.65 | 1.28(0.46,3.57) | 0.63 |
| **Combinations of type and BP control level** | | | | | | | |
| Hypertension and well controlled BP | 3/24 (12.50) | Reference | | Reference | | Reference | |
| Hypertension and poorly controlled BP | 9/47 (19.15) | 1.53(0.41,5.66) | 0.52 | 1.32(0.34,5.11) | 0.69 | 1.26(0.31,5.13) | 0.74 |
| Preeclampsia and well controlled BP | 2/9 (22.22) | 1.78(0.3,10.64) | 0.53 | 1.7(0.24,12.02) | 0.60 | 1.52(0.21,11.18) | 0.68 |
| Preeclampsia and poorly controlled BP | 14/56 (25.00) | 2(0.57,6.96) | 0.28 | 1.75(0.45,6.75) | 0.42 | 1.75(0.45,6.86) | 0.42 |

Abbreviations: HDP, hypertensive disorder of pregnancy; RR, relative risk; BP, blood pressure.

Model 1: Crude. Model 2: Adjusted for maternal age, paternal age, maternal educational level, maternal residence, household annual income, mode of conception, singleton, offspring sex, and offspring age. Model 3: Adjusted for maternal age, paternal age, maternal educational level, maternal residence, household annual income, mode of conception, singleton, offspring sex, offspring age, mode of delivery, gestational week at birth, birth weight, and offspring refractive error.

© 2024 Zhu H et al. *JAMA Network Open*.

**eFigure.** Directed Acyclic Graph Documenting Assumptions About the Association Between Covariates, Exposure, and Outcome[a]

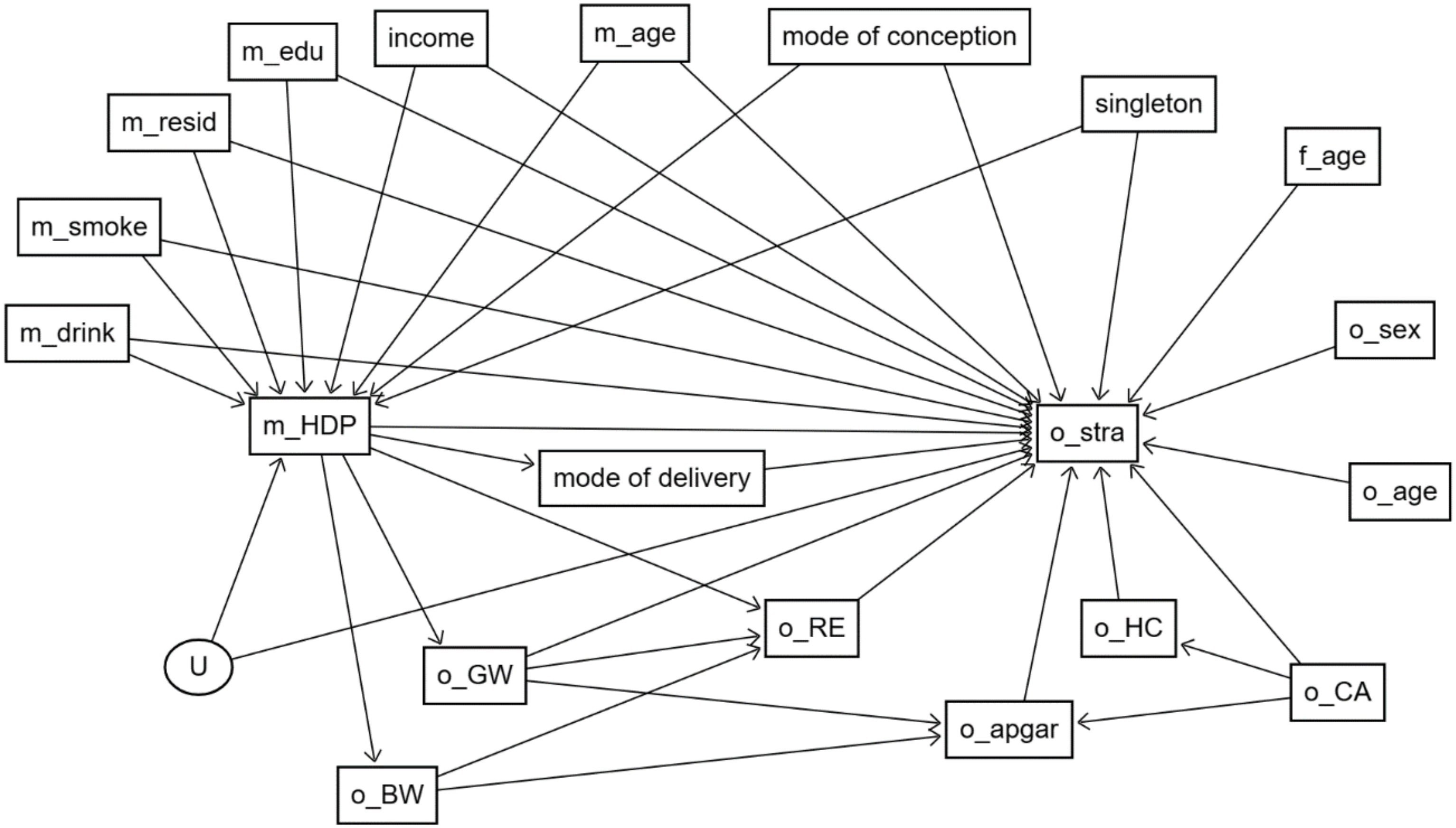


[a] m_HDP: maternal HDP; o_stra: offspring strabismus; m_drink: maternal alcohol consumption; m_smoke: maternal smoking; m_resid: maternal residence; m_age: maternal age; m_edu: maternal education; f_age: paternal age; o_sex: offspring sex; o_age: offspring age; o_CA: offspring congenital abnormality; o_agpar: offspring Apgar score; o_HC: offspring head circumference; o_RE: offspring refractive error; o_GW: offspring gestational week; o_BW: offspring birth weight; U: unmeasured variable

© 2024 Zhu H et al. *JAMA Network Open*.
