# Supplementary material for: Maternal Hypertensive Disorder in Pregnancy and Childhood Strabismus in Offspring
Source: JAMA Netw Open. 2024 Jul 22;7(7):e2423946. doi: 10.1001/jamanetworkopen.2024.23946 (PMC11265127; doi:10.1001/jamanetworkopen.2024.23946)
Supplement: Supplement 2. — Nonauthor Collaborators [file jamanetwopen-e2423946-s002.pdf]

Supplemental Online Content: Nonauthor Collaborators
*First name, last name, and suffix (if applicable) are required and will appear in PubMed.

*Group Name(s): The China National Birth Cohort Study Group							
***First Name and Middle Initial(s)**	***Last Name**	***Suffix (eg, Jr, III)**	Academic Degrees	Institution	Location (city, state/province, country)	Role or Contribution, eg, chair, principal investigator	Group (if more than 1 Group listed in the byline) and/or Subgroup (eg, Steering Committee)
Hongbing	Shen		MD,PhD	Nanjing Medical University	Nanjing, Jiangsu, China		
Yankai	Xia		MD,PhD	Nanjing Medical University	Nanjing, Jiangsu, China		
Jiong	Li		MD,PhD	Nanjing Medical University	Nanjing, Jiangsu, China		
Chuncheng	Lu		MD,PhD	Nanjing Medical University	Nanjing, Jiangsu, China		
Yang	Zhao		MD,PhD	Nanjing Medical University	Nanjing, Jiangsu, China		
Wei	Wu		MD,PhD	Nanjing Medical University	Nanjing, Jiangsu, China		
Minjian	Chen		MD,PhD	Nanjing Medical University	Nanjing, Jiangsu, China		
Shanshan	Geng		MD,PhD	Nanjing Medical University	Nanjing, Jiangsu, China		
Ye	Ding		MD,PhD	Nanjing Medical University	Nanjing, Jiangsu, China		
Lei	Huang		MD,PhD	Nanjing Medical University	Nanjing, Jiangsu, China		
Shiyao	Tao		MD,PhD	Nanjing Medical University	Nanjing, Jiangsu, China		
Yuanyan	Dou		MD,PhD	Nanjing Medical University	Nanjing, Jiangsu, China		
Shuifang	Lei		MD,PhD	Nanjing Medical University	Nanjing, Jiangsu, China		
Kang	Ke		MD,PhD	Nanjing Medical University	Nanjing, Jiangsu, China		
Yanjie	Zhang		MD,PhD	Nanjing Medical University	Nanjing, Jiangsu, China		
Yiqun	Xu		MD,PhD	Nanjing Medical University	Nanjing, Jiangsu, China		
Jinghan	Wang		MD,PhD	Nanjing Medical University	Nanjing, Jiangsu, China		
Xianxian	Zhu		MD	Nanjing Medical University	Nanjing, Jiangsu, China		
Huixin	Xue		MD	Nanjing Medical University	Nanjing, Jiangsu, China		
Ming	Gan		MD	Nanjing Medical University	Nanjing, Jiangsu, China		
Shuxin	Xiao		MD	Nanjing Medical University	Nanjing, Jiangsu, China		
Weiting	Wang		MD	Nanjing Medical University	Nanjing, Jiangsu, China		
Di	Pi		MD	Nanjing Medical University	Nanjing, Jiangsu, China		
Chang	Wang		MD	Nanjing Medical University	Nanjing, Jiangsu, China		
Haowen	Zhang		MD	Nanjing Medical University	Nanjing, Jiangsu, China		
Bo	Yang		MD	Nanjing Medical University	Nanjing, Jiangsu, China		
Yufan	Jin		MD	Nanjing Medical University	Nanjing, Jiangsu, China		
Xiao	Zhang		MD	Nanjing Medical University	Nanjing, Jiangsu, China		
Yongbin	Xiong		MD	Nanjing Medical University	Nanjing, Jiangsu, China		
Xin	Zou		MD	Nanjing Medical University	Nanjing, Jiangsu, China		

Supplemental Online Content: Nonauthor Collaborators
*First name, last name, and suffix (if applicable) are required and will appear in PubMed.

***First Name and Middle Initial(s)**	***Last Name**	***Suffix (eg, Jr, III)**	Academic Degrees	Institution	Location (city, state/province, country)	Role or Contribution, eg, chair, principal investigator	Group (if more than 1 Group listed in the byline) and/or Subgroup (eg, Steering Committee)
Zheng	Yong		MD	Nanjing Medical University	Nanjing, Jiangsu, China		
Shuting	Wu		MD	Nanjing Medical University	Nanjing, Jiangsu, China		
Yue	Qu		MD	Nanjing Medical University	Nanjing, Jiangsu, China		
Qin	Wang		MD	Nanjing Medical University	Nanjing, Jiangsu, China		
Jie	Chen		MD	Nanjing Medical University	Nanjing, Jiangsu, China		
Ganchong	Liao		MD	Nanjing Medical University	Nanjing, Jiangsu, China		
Liya	Pang		MD	Nanjing Medical University	Nanjing, Jiangsu, China		
Dan	Huang		Ph.D	The First Affiliated Hospital with Nanjing Medical University	Nanjing, Jiangsu, China		
Zhujun	Fu		Ph.D	Children's Hospital of Nanjing Medical University	Nanjing, Jiangsu, China		
Zijin	Wang		Ph.D	The First Affiliated Hospital with Nanjing Medical University	Nanjing, Jiangsu, China		
Yue	Wang		Ph.D	Nanjing First Hospital with Nanjing Medical University	Nanjing, Jiangsu, China		
Rui	Li		Ph.D	Nanjing First Hospital with Nanjing Medical University	Nanjing, Jiangsu, China		
Haohai	Tong		M.M.	The Second Affiliated Hospital with Zhejiang University School of Medicine	Hangzhou, Zhejiang, China		
Shu	Han		M.M.	Children's Hospital of Soochow University	Suzhou, Jiangsu, China		
Xiaoyan	Zhao		M.M.	Changzhou NO.2 People's Hospital with Nanjing Medical University	Changzhou, Jiangsu, China		
Shiya	Shen		M.M.	The First Affiliated Hospital with Nanjing Medical University	Nanjing, Jiangsu, China		
Yun	Wang		M.M.	Woman's Hospital of Nanjing Medical University	Nanjing, Jiangsu, China		
Wen	Yan		M.M.	Yancheng First Hospital Affiliated Hospital of Nanjing University Medical School	Yancheng, Jiangsu,China		

Supplemental Online Content: Nonauthor Collaborators
*First name, last name, and suffix (if applicable) are required and will appear in PubMed.

*First Name and Middle Initial(s)	*Last Name	*Suffix (eg, Jr, III)	Academic Degrees	Institution	Location (city, state/province, country)	Role or Contribution, eg, chair, principal investigator	Group (if more than 1 Group listed in the byline) and/or Subgroup (eg, Steering Committee)
Xiaoxiao	Li		M.M.	Children's Hospital of Soochow University	Suzhou, Jiangsu, China		
Andi	Zhao		Ph.D	The First Affiliated Hospital with Nanjing Medical University	Nanjing, Jiangsu, China		
Danni	Chen		M.M.	Children's Hospital with Nanjing Medical University	Nanjing, Jiangsu, China		
Lei	Liu		M.M.	Jiangsu College Of Nursing	Huaian, Jiangsu, China		
Wei	Guo		Ph.D	The First Affiliated Hospital with Nanjing Medical University	Nanjing, Jiangsu, China		
Yingxiao	Qian		M.M.	Changzhou Health Vocational Technology College	Changzhou, Jiangsu, China		
Lei	Hua		M.M.	Eastern Theater General Hospital, Qinhuai District Medical Area	Nanjing, Jiangsu, China		
Qi	Yan		M.M.	The First Affiliated Hospital with Nanjing Medical University	Nanjing, Jiangsu, China		
Xiaoxia	Zuo		M.M.	The First Affiliated Hospital with Nanjing Medical University	Nanjing, Jiangsu, China		
Tong	Zhang		M.M.	The First Affiliated Hospital with Nanjing Medical University	Nanjing, Jiangsu, China		
Ziyi	Ni		M.M.	The First Affiliated Hospital with Nanjing Medical University	Nanjing, Jiangsu, China		
Yu	Jing		M.M.	The First Affiliated Hospital with Nanjing Medical University	Nanjing, Jiangsu, China		
Tiantian	Zhou		M.M.	The First Affiliated Hospital with Nanjing Medical University	Nanjing, Jiangsu, China		
Xiaoqi	Zhu		M.M.	The First Affiliated Hospital with Nanjing Medical University	Nanjing, Jiangsu, China		
Chenyu	Zhou		M.M.	The First Affiliated Hospital with Nanjing Medical University	Nanjing, Jiangsu, China		
Yuting	Zhang		B.M.	Nanjing Medical University	Nanjing, Jiangsu, China		

Supplemental Online Content: Nonauthor Collaborators
*First name, last name, and suffix (if applicable) are required and will appear in PubMed.

***First Name and Middle Initial(s)**	***Last Name**	***Suffix (eg, Jr, III)**	Academic Degrees	Institution	Location (city, state/province, country)	Role or Contribution, eg, chair, principal investigator	Group (if more than 1 Group listed in the byline) and/or Subgroup (eg, Steering Committee)
Yunfan	Shi		B.M.	The First Affiliated Hospital with Nanjing Medical University	Nanjing, Jiangsu, China		
Yuhong	Feng		B.M.	The First Affiliated Hospital with Nanjing Medical University	Nanjing, Jiangsu, China		
Chuxuan	Zhang		B.M.	The First Affiliated Hospital with Nanjing Medical University	Nanjing, Jiangsu, China		
Haodong	Chi		B.M.	Nanjing Medical University	Nanjing, Jiangsu, China		
Fanbo	Sun		B.M.	Nanjing Medical University	Nanjing, Jiangsu, China		
Jian	Cui		B.M.	Nanjing Medical University	Nanjing, Jiangsu, China		
Yan	Gao		B.M.	Nanjing Medical University	Nanjing, Jiangsu, China		
Xinyuan	Yao		B.M.	Nanjing Medical University	Nanjing, Jiangsu, China		
Meng	Li		B.M.	Nanjing Medical University	Nanjing, Jiangsu, China		
Jiahao	Si		B.M.	Nanjing Medical University	Nanjing, Jiangsu, China		
Shasha	Xu		B.M.	Nanjing Medical University	Nanjing, Jiangsu, China		
Xi	Chen		B.M.	Nanjing Medical University	Nanjing, Jiangsu, China		
Jiyu	Zhang		B.M.	Nanjing Medical University	Nanjing, Jiangsu, China		
Xinyu	Zheng		B.M.	Nanjing Medical University	Nanjing, Jiangsu, China		
Yelongzi	Cao		B.M.	Nanjing Medical University	Nanjing, Jiangsu, China		
Xiaodong	Shao		B.M.	Nanjing Medical University	Nanjing, Jiangsu, China		
Shuning	Liu		B.M.	Nanjing Medical University	Nanjing, Jiangsu, China		
Yuxi	Chen		B.M.	Nanjing Medical University	Nanjing, Jiangsu, China		
Qin	Shu		B.M.	Nanjing Medical University	Nanjing, Jiangsu, China		
Shiding	Li		B.M.	Nanjing Medical University	Nanjing, Jiangsu, China		
Jingsong	Dang		B.M.	Nanjing Medical University	Nanjing, Jiangsu, China		
Tao	Zhang		B.M.	Nanjing Medical University	Nanjing, Jiangsu, China		
Ranran	Ding		B.M.	Nanjing Medical University	Nanjing, Jiangsu, China		
Yuemin	Wu		B.M.	Nanjing Medical University	Nanjing, Jiangsu, China		
Liyuan	Wang		B.M.	Nanjing Medical University	Nanjing, Jiangsu, China		
Ningjing	Zhao		B.M.	Nanjing Medical University	Nanjing, Jiangsu, China		
Jiajia	Yang		B.M.	Nanjing Medical University	Nanjing, Jiangsu, China		
Shuoyang	Zhao		B.M.	Nanjing Medical University	Nanjing, Jiangsu, China		

Supplemental Online Content: Nonauthor Collaborators
*First name, last name, and suffix (if applicable) are required and will appear in PubMed.

*First Name and Middle Initial(s)	*Last Name	*Suffix (eg, Jr, III)	Academic Degrees	Institution	Location (city, state/province, country)	Role or Contribution, eg, chair, principal investigator	Group (if more than 1 Group listed in the byline) and/or Subgroup (eg, Steering Committee)
Yichao	Qian		B.M.	Nanjing Medical University	Nanjing, Jiangsu, China		
Yao	Lu		B.M.	Nanjing Medical University	Nanjing, Jiangsu, China		
Yue	Zhu		B.M.	Nanjing Medical University	Nanjing, Jiangsu, China		
Fanfei	Ma		B.M.	Nanjing Medical University	Nanjing, Jiangsu, China		
Changqin	Zhang		B.M.	Nanjing Medical University	Nanjing, Jiangsu, China		
Yuxiao	Xu		B.M.	Nanjing Medical University	Nanjing, Jiangsu, China		
Yan	Zhu		B.M.	Nanjing Medical University	Nanjing, Jiangsu, China		
Weiyi	Dong		B.M.	Nanjing Medical University	Nanjing, Jiangsu, China		
Mengqi	Dong		B.M.	Nanjing Medical University	Nanjing, Jiangsu, China		
Zhitong	Li		B.M.	Nanjing Medical University	Nanjing, Jiangsu, China		
Jing	Sun		B.M.	Nanjing Medical University	Nanjing, Jiangsu, China		
Yuxiang	Yi		B.M.	Nanjing Medical University	Nanjing, Jiangsu, China		
Hongxue	Fu		B.M.	Nanjing Medical University	Nanjing, Jiangsu, China		
Jie	Dou		B.M.	Nanjing Medical University	Nanjing, Jiangsu, China		
Wanting	Gao		B.M.	Nanjing Medical University	Nanjing, Jiangsu, China		
Zhuorong	Li		B.M.	Nanjing Medical University	Nanjing, Jiangsu, China		
Leyan	Sun		B.M.	Nanjing Medical University	Nanjing, Jiangsu, China		
Lan	Tang		B.M.	Nanjing Medical University	Nanjing, Jiangsu, China		
Xinyan	Xu		B.M.	Nanjing Medical University	Nanjing, Jiangsu, China		
Jingwen	Yang		B.M.	Nanjing Medical University	Nanjing, Jiangsu, China		
Ping	Xue		B.M.	Nanjing Medical University	Nanjing, Jiangsu, China		
Shengxiang	Huang		B.M.	Nanjing Medical University	Nanjing, Jiangsu, China		
Yidan	Xia		B.M.	Nanjing Medical University	Nanjing, Jiangsu, China		
Sirui	Gu		B.M.	Nanjing Medical University	Nanjing, Jiangsu, China		
Yaxin	Chen		B.M.	Nanjing Medical University	Nanjing, Jiangsu, China		
Yizheng	Zhang		B.M.	Nanjing Medical University	Nanjing, Jiangsu, China		
Qi	Chen		B.M.	Nanjing Medical University	Nanjing, Jiangsu, China		
Ruize	Wu		B.M.	Nanjing Medical University	Nanjing, Jiangsu, China		
Anqi	Shi		B.M.	Nanjing Medical University	Nanjing, Jiangsu, China		
Yujie	Jiang		B.M.	Nanjing Medical University	Nanjing, Jiangsu, China		
Rui	Xu		B.M.	Nanjing Medical University	Nanjing, Jiangsu, China		

Supplemental Online Content: Nonauthor Collaborators
*First name, last name, and suffix (if applicable) are required and will appear in PubMed.

***First Name and Middle Initial(s)**	***Last Name**	***Suffix (eg, Jr, III)**	Academic Degrees	Institution	Location (city, state/province, country)	Role or Contribution, eg, chair, principal investigator	Group (if more than 1 Group listed in the byline) and/or Subgroup (eg, Steering Committee)
Linting	Mei		B.M.	Nanjing Medical University	Nanjing, Jiangsu, China		
Xuexian	Zhu		B.M.	Nanjing Medical University	Nanjing, Jiangsu, China		
Yiting	Wang		B.M.	Nanjing Medical University	Nanjing, Jiangsu, China		
Xianyue	Liang		B.M.	Nanjing Medical University	Nanjing, Jiangsu, China		
Jing	Xu		B.M.	Nanjing Medical University	Nanjing, Jiangsu, China		
Zheng	Zhang		B.M.	Nanjing Medical University	Nanjing, Jiangsu, China		
Chengfei	Xu		B.M.	Nanjing Medical University	Nanjing, Jiangsu, China		
Jin	Liu		B.M.	Nanjing Medical University	Nanjing, Jiangsu, China		
Yucao	Wang		B.M.	Nanjing Medical University	Nanjing, Jiangsu, China		
Jinghan	Liang		B.M.	Nanjing Medical University	Nanjing, Jiangsu, China		
Jiaxu	Li		B.M.	Nanjing Medical University	Nanjing, Jiangsu, China		
Junyi	You		B.M.	Nanjing Medical University	Nanjing, Jiangsu, China		
Zhiying	Lin		B.M.	Nanjing Medical University	Nanjing, Jiangsu, China		
Wenjie	Zhou		B.M.	Nanjing Medical University	Nanjing, Jiangsu, China		
Zihan	Yin		B.M.	Nanjing Medical University	Nanjing, Jiangsu, China		
Cheng	Sun		B.M.	Nanjing Medical University	Nanjing, Jiangsu, China		
Difei	Zuo		B.M.	Nanjing Medical University	Nanjing, Jiangsu, China		
Ruini	Zhu		B.M.	Nanjing Medical University	Nanjing, Jiangsu, China		
Qikun	Deng		B.M.	Nanjing Medical University	Nanjing, Jiangsu, China		
Yujiao	Shi		B.M.	Nanjing Medical University	Nanjing, Jiangsu, China		
Yujie	Wang		B.M.	Nanjing Medical University	Nanjing, Jiangsu, China		
Zhen	Wang		B.M.	Nanjing Drum Tower Eye Clinic of Children and Adolescents	Nanjing, Jiangsu, China		
Xiaoyu	Fan		B.M.	Nanjing Drum Tower Eye Clinic of Children and Adolescents	Nanjing, Jiangsu, China		
Xinying	Li		B.M.	Nanjing Drum Tower Eye Clinic of Children and Adolescents	Nanjing, Jiangsu, China		
