# Supplementary material for: Maternal Hypertensive Disorder in Pregnancy and Childhood Strabismus in Offspring
Source: JAMA Netw Open. 2024 Jul 22;7(7):e2423946. doi: 10.1001/jamanetworkopen.2024.23946 (PMC11265127; doi:10.1001/jamanetworkopen.2024.23946)
Supplement: Supplement 3. — Data Sharing Statement [file jamanetwopen-e2423946-s003.pdf]

Data Sharing Statement

Zhu. Maternal Hypertensive Disorder in Pregnancy and Childhood Strabismus in Offspring. *JAMA Netw Open*. Published July 22, 2024. doi:10.1001/jamanetworkopen.2024.23946

Data
Data available: No
